# Aligned Nanostructures Resolve Zn^2+^ Transport Bottlenecks via Interfacial Kinetics–Diffusion Coupling in Aqueous Zinc‐Ion Batteries

**DOI:** 10.1002/advs.202512691

**Published:** 2025-11-20

**Authors:** Juyeon Han, Eunha Seo, Subeen Park, Se Hun Lee, Jeeyoung Yoo

**Affiliations:** ^1^ School of Energy Engineering Kyungpook National University Daegu 41566 Republic of Korea; ^2^ Advanced Institute of Convergence Technology Seoul National University Suwon 16229 Republic of Korea

**Keywords:** interfacial kinetics, ion transport bottlenecks, Multiphysics simulation, operando‐analysis, vanadium‐based cathode

## Abstract

Aqueous zinc‐ion batteries (AZIBs) have garnered significant attention as a safe and cost‐effective alternative to lithium‐ion batteries for grid‐scale energy storage. However, their performance is hindered by sluggish Zn^2+^ diffusion within the cathode and structural degradation. While pre‐intercalation strategies have demonstrated improvements in electrochemical performance, the comprehensive understanding between synthesis‐driven evolution, Zn^2+^ diffusion, and interphase kinetics remains underexplored. Herein, it is investigated how synthesis time influences the structure and morphology of K_2_V_6_O_16_·nH_2_O cathodes, as well as their Zn^2+^ diffusion and charge transfer kinetics. By coupling *operando‐* electrochemical impedance spectroscopy (EIS) and COMSOL simulation, that interfacial Zn^2^⁺ accumulation, induced by limited solid‐state diffusion within the cathode, leads to pronounced transport bottlenecks—despite sufficient charge‐transfer kinetics is identified. This imbalance distorts the Zn^2+^ flux directionality and creates spatial heterogeneity in ion transport. Notably, these bottlenecks are effectively alleviated by 1D nanostructured architectures, which promote continuous ion transport and facilitate interfacial reaction kinetics. Consequently, K_2_V_6_O_16_·nH_2_O exhibits a tenfold increase in Zn^2+^ diffusivity and 97.26% capacity retention over 5000 cycles. These findings offer valuable insights into the rational design of high‐performance AZIB cathodes through synthesis‐driven structural control.

## Graphical abstract

It is revealed that limited solid‐state Zn^2^⁺ diffusion within the cathode leads to interfacial Zn^2^⁺ accumulation, forming a transport bottleneck that distorts Zn^2^⁺ flux directionality. Through advanced analytical techniques, including in situ electrochemical impedance spectroscopy (EIS), distribution of relaxation time (DRT) analysis, and COMSOL simulation, this study provides a detailed evaluation of synthesis‐driven evolution enhances electrochemical performance.

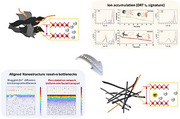

## 1 Introduction

As the global energy landscape shifts away from fossil fuels, the development of high‐performance energy storage technologies is crucial for sustainable energy integration.^[^ ^1^ ^]^ Lithium‐ion batteries (LIBs) are widely adopted due to their high energy density and efficiency.^[^ ^2^, ^3^ ^]^ Yet, concerns over volatile organic electrolytes and potential lithium dendrite formation pose significant safety risks—especially in large‐scale or long‐term applications. To address these limitations, aqueous Zinc ion batteries (AZIBs) have gained significant attention as a next‐generation energy storage system, leveraging the multivalent nature of Zn^2+^ to achieve a high theoretical capacity (820 mAh g^−1^). Their inherently non‐flammable aqueous electrolytes mitigate the safety risks associated with LIBs, while their cost‐effectiveness and environmental sustainability make them attractive for large‐scale applications.^[^ ^4^ ^]^

Despite these advantages, AZIBs face several critical challenges that hinder their widespread application, primarily stemming from the instability of the Zn metal/electrolyte interphase and the sluggish Zn^2+^ diffusion within the cathode.^[^ ^5^ ^]^ Dendrite formation and side reactions at the Zn metal anode often lead to internal short. To mitigate these shortcomings, researchers have developed strategies such as coating modifications, separator engineering and electrolyte optimization, yielding notable improvements in anode stability and overall battery performance.^[^ ^6^, ^7^, ^8^, ^9^, ^10^ ^]^ Meanwhile, strong electrostatic interactions arising from the multivalent nature of Zn^2+^ induce pronounced polarization, significantly restricting Zn^2+^ diffusion and limiting the battery's performance.^[^ ^11^, ^12^ ^]^ Therefore, developing cathode materials that enable improved Zn^2+^ transport and structural stability is crucial for achieving reliable long‐term cycling. A variety of cathode candidates, including manganese‐based compounds, vanadium‐based compounds, and Prussian blue analogues, have been extensively explored.^[^ ^13^, ^14^, ^15^, ^16^ ^]^ Nonetheless, several intrinsic limitations—such as poor electronic conductivity, sluggish Zn^2+^ diffusion, dissolution of active materials into the electrolyte, detrimental phase transitions, and structural collapse—continue to hinder the practical applications of these cathode materials.

Among these cathode candidates, vanadium‐based oxides, particularly V_2_O_5_, have attracted considerable attention owing to their layered structure, multiple redox states, and high theoretical capacity.^[^ ^17^, ^18^, ^19^ ^]^ However, their electrochemical performance remains limited by inherently narrow interlayer spacing (≈4.3 Å in the orthorhombic phase, space group: Pmn2), leading to sluggish Zn^2+^ diffusion and severe polarization under cycling conditions.^[^ ^20^ ^]^ To overcome these challenges, pre‐intercalation of alkali metal ions (Li^+^, Na^+^, K^+^, etc.) and water molecules has been explored as an effective strategy to expand interlayer spacing, mitigate electrostatic interaction, and lower the insertion energy barrier of Zn^2+^.^[^ ^21^, ^22^, ^23^ ^]^ This pre‐intercalation strategy modifies interlayer bonding and enables structural rearrangement, promoting layer fragmentation and subsequent reassembly into nanostructured architectures. These nanostructures facilitate Zn^2+^ diffusion by providing continuous transport channels, reducing interfacial resistance, and mitigating structural degradation over prolonged cycling.^[^ ^14^, ^21^ ^]^

While this strategy improves performance, a fundamental mechanistic understanding—particularly how synthesis‐driven structural evolution governs Zn^2+^ diffusion pathways and interfacial charge‐transfer kinetics—is lacking. Prior *operando* studies have emphasized lattice or phase changes rather than the ion dynamics, leaving the distinction between bulk Zn^2+^ diffusion and interfacial kinetics underexplored. In this study, through controlled synthesis of K_2_V_6_O_16_·nH_2_O (KVO) electrodes—which undergo a synthesis‐driven transformation into 1D nanofibers—we quantitatively monitor the dynamic behavior of Zn^2+^ diffusivity and charge‐transfer kinetics. By coupling *operando*‐electrochemical impedance spectroscopy (EIS) and distribution of relaxation times (DRT) analysis, we reveal that key kinetic barriers that govern electrochemical performance originate from solid‐state Zn^2+^ diffusion within the cathode. These diffusion limitations lead to local ion accumulation at the electrode/electrolyte interphase, which in turn distorts the Zn^2+^ flux directionality and creates spatial heterogeneity in ion transport— a mechanism further corroborated by COMSOL simulations.

Consequently, 1D nanofiber KVO architecture substantially alleviates this bottleneck, achieving a Zn^2+^ diffusion coefficient of 1.41 × 10^−9^ cm^2^ s^−1^ and delivering stable performance over 5000 cycles with 97.26% capacity retention. These insights establish a mechanistic foundation for mitigating interfacial bottlenecks in AZIBs and offer structural design guidelines to enhance ion transport.

## 2 Results and Discussion

### 2.1 Structural and Morphological Characterization

**Figure 1:**
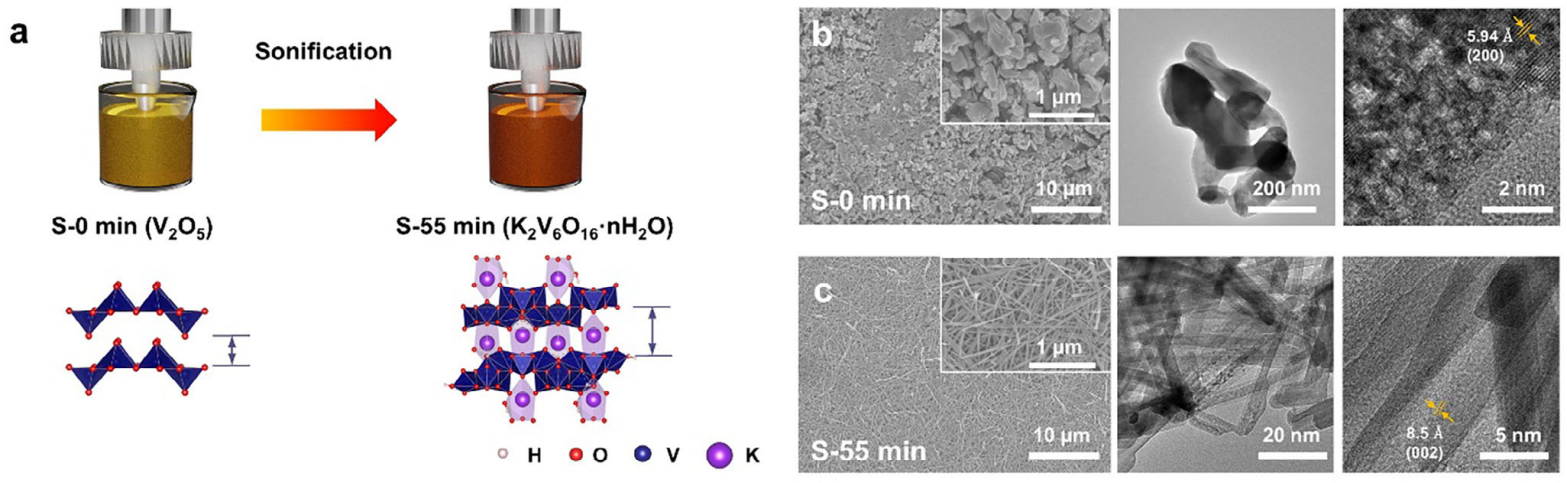
Synthesis‐driven structural transformation of KVO. a) Schematic illustration of the sonochemical synthesis process. b,c) SEM, TEM images of the S‐0 and S‐55 min.

The KVO was synthesized via a sonochemical process, during which the precursor solution gradually changed from yellow to dark red, visually indicating a progressive phase transformation (**Figure** **1**a). At the initial stage (S‐0 min), VO_5_ square pyramids are arranged in densely stacked layers, which restrict Zn^2+^ intercalation and limit reversible capacity.^[^ ^24^, ^25^ ^]^ As the synthesis progresses to S‐55 min, the material undergoes a phase transition to KVO, where K⁺ occupy interlayer sites and stabilizes an expanded framework. This phase consists of both VO_5_ pyramids and VO_6_ distorted octahedra, with K⁺ preferentially coordinating near partially negative oxygen atoms in terminal V = O bonds.

This structural transformation plays a crucial role in electrochemical performance. Vanadium‐based cathodes exhibit diverse vanadium‐oxygen polyhedral configurations, including tetrahedral, square pyramidal, and octahedral units, with oxidation states ranging from +5 to +3 (Figure S1, Supporting Information).^[^ ^26^, ^27^ ^]^ The VO_5_ square pyramidal configuration is inherently unstable in aqueous environments due to the susceptibility of exposed vanadium sites to hydrolysis. In contrast, VO_6_ octahedral coordination provides superior structural stability and enables a reversible V^5+^/V^3+^ redox transition with minimal lattice distortion.^[^ ^28^ ^]^ Supporting this observation, ICP‐OES analysis (Figure S2, Supporting Information) confirms that only S‐55 min suppressed vanadium dissolution, highlighting the improved aqueous stability conferred by VO_6_ coordination. This vanadium dissolution leads to electrolyte acidification, further destabilizing the electrode/electrolyte interphase.

SEM and TEM images (Figure 1b,c) reveal a pronounced morphological transformation from dense, plate‐like particles (S‐0 min) to well‐defined 1D nanofibers (S‐55 min). This transformation is driven by K^+^ intercalation, layer rearrangement, and oxidative effects from SO_4_^2−^ radicals, which collectively guide anisotropic crystal growth and enhance ion transport pathways. TEM analysis further confirms a significant expansion in interlayer distance, increasing from 5.94 Å (200) to 8.5 Å (002). The continuous evolution of morphology and composition throughout the synthesis process is further supported by SEM, TEM, and EDS data at multiple time points, as shown in Figures S3–S5 (Supporting Information). The (002) plane corresponding to the KVO first appears at S‐10 min, indicating the onset of phase transformation. Notably, this morphological transition to a 1D nanofibrous architecture significantly increases the specific surface area, as confirmed by BET analysis (Figure S6 and Table S1, Supporting Information). The surface area increases dramatically from 7.4 to 48.3 m^2^ g^−1^ at S‐55 min, providing more electrochemically active sites and facilitating Zn^2+^ diffusion.

**Figure 2:**
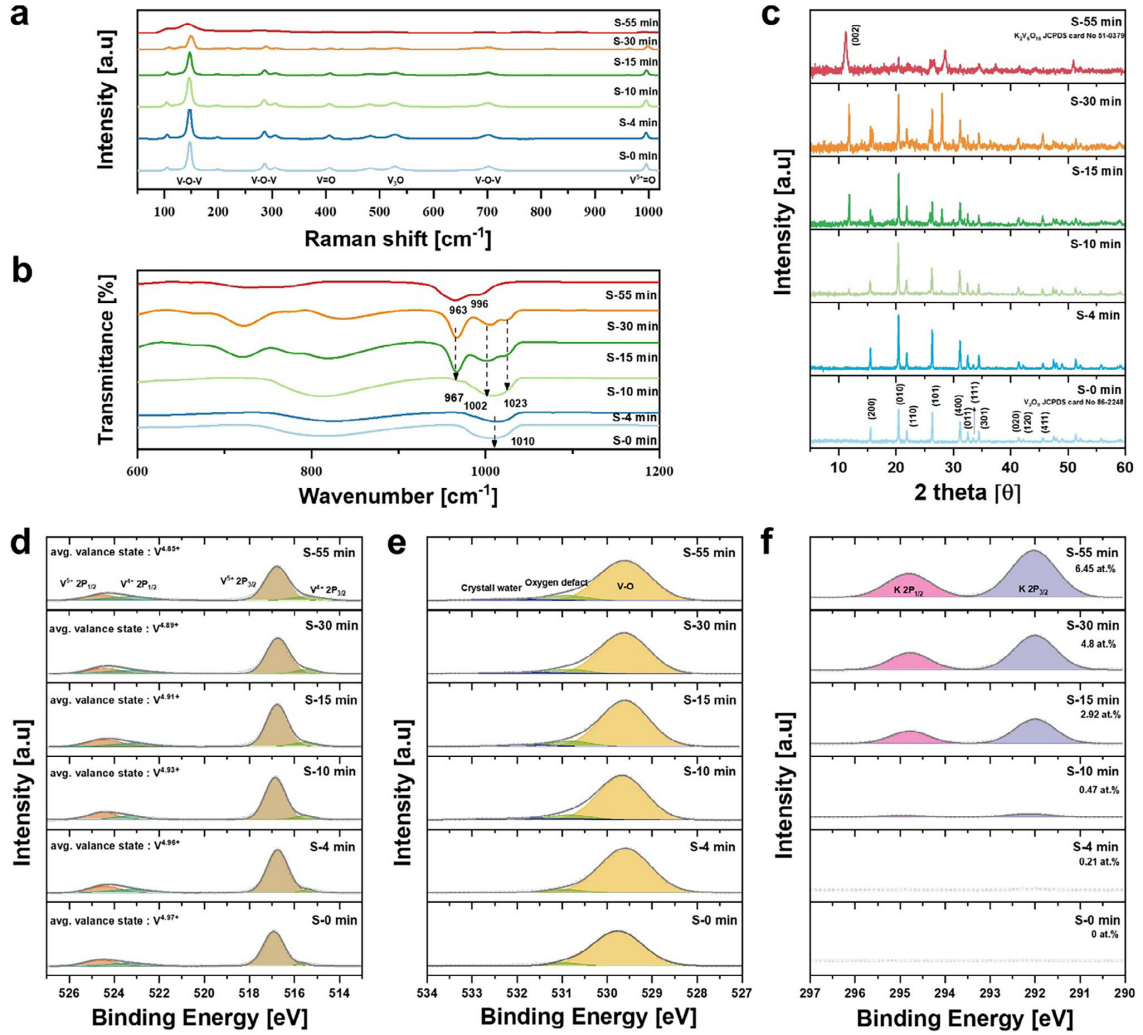
Synthesis‐time‐dependent structural and chemical evolution of KVO cathodes. a) Raman spectra b) FT–IR spectra. c) XRD patterns illustrating the structural transition from orthorhombic V_2_O_5_ (S‐0 min) to monoclinic KVO (S‐55 min). d–f) XPS analysis of V 2p, O 1s, and K 2p regions.

Raman spectroscopy was performed to investigate structural evolution as a function of synthesis time (**Figure** **2**a). As synthesis time increases, the Raman peaks progressively broaden and diminish in intensity (Figure S7, Supporting Information), indicating increased structural disorder and altered interlayer interactions. Furthermore, in the 600–800 cm^−1^ region, additional peaks emerge at 772.4 and 865.0 cm^−1^, corresponding to the V–O stretching vibrations associated with VO_6_.^[^ ^29^, ^30^, ^31^ ^]^ These vibrational fingerprints reflect a local coordination transition, signaling the formation of hydrated layered vanadate. Such reconfiguration of the V–O framework is expected to reduce the electrostatic interaction between Zn^2+^ and the host lattice, thereby lowering the insertion barrier and facilitating more efficient ion transport.

This structural evolution is further supported by FT‐IR analysis, which provides complementary insights into the bonding environment of vanadium oxides (Figure 2b). The strong peak at 1010 cm^−1^ in S‐0 min corresponds to the V = O stretching vibration. After S10 min, this band splits into two peaks at 1002 and 1023 cm^−1^, attributed to two inequivalent V^5+^ = O groups of distorted octahedral and square pyramidal units, respectively.^[^ ^32^, ^33^ ^]^ Additionally, a peak at 967 cm^−1^, which first appears at S‐10 min, corresponds to V^4+^ = O stretching of distorted octahedra.^[^ ^34^ ^]^ This observation suggests that the structural transformation from V_2_O_5_ to KVO begins at S‐10 min. Notably, at S‐55 min, the V = O stretching vibration is red‐shifted to 996 and 963 cm^−1^, indicating a progressive reduction of V^5+^ to V^4+^ and continuous electronic structure modification during synthesis. The hydration‐related coordination change is further confirmed by TGA analysis (Figure S8, Supporting Information), which shows a 5.6% weight loss at S‐55 min—consistent with the presence of intercalated water molecules within the V–O framework.

The XRD pattern at S‐0 min corresponds to orthorhombic α‐V_2_O_5_ (JCPDS No. 86‐2248; a  =  11.503 Å, b  =  4.369 Å, and c  =  3.557 Å) (Figure 2c).^[^ ^35^ ^]^ As the synthesis progresses, the structure undergoes a gradual phase transition into KVO, adopting a monoclinic phase (JCPDS No. 51‐0379) with lattice parameters of a  =  12.30 Å, b  =  3.60 Å, and c  =  16.02 Å.^[^ ^36^ ^]^ A key structural hallmark of this transformation is the emergence of a new peak at 2θ = 11.8°, corresponding to the (002) plane of KVO, which first appears in S‐10 min.^[^ ^37^ ^]^ As shown in Figure S9 (Supporting Information), the (002) diffraction peak shifts to a lower angle (2θ = 11.23°) upon reaction completion (S‐55 min), corresponding to an increase in interlayer spacing from 0.749 to 0.787 nm. This increase in interlayer spacing indicates that K^+^ intercalate into the V−O layers, resulting in lattice expansion, which enhances structural stability and promotes more efficient Zn^2+^ diffusion.

To further investigate elemental composition and oxidation state variations over synthesis time, XPS analysis was conducted. The V 2p spectrum (Figure 2d) shows V^4+^ (515.6/523.2 eV) and V^5+^ (516.9/524.4 eV), with the average vanadium oxidation state decreasing from 4.97 to 4.85, consistent with EPR results (Figure S10, Supporting Information).^[^ ^38^, ^39^ ^]^ The O 1s spectrum (Figure 2e) reveals distinct peaks corresponding to oxygen defects (530.9 eV) and V–O bonds (529.6 eV). The oxygen vacancy ratio steadily increases from 3.7% at S‐0 min to 11.9% at S‐55 min. Notably, a new peak at 532.1 eV, associated with water insertion, emerges at S‐10 min, indicating hydration effects during the reaction. In the K 2p spectra, the atomic percentage of K⁺ progressively increased with synthesis time, reaching 6.45% at S‐55 min (Figure 2f). These electronic structure modifications are accompanied by a visible color change (Figure S11, Supporting Information), reflecting the partial reduction of V^5+^ to V^4+^ and a corresponding narrowing of the band gap.^[^ ^40^, ^41^ ^]^

Consequently, K^+^ insertion induces structural phase transition along with morphological reorganization. The resulting interconnected 1D pathways, together with the expanded interlayer spacing and improved structural stability, facilitate continuous Zn^2+^ transport and suppress lattice distortion during cycling. Such structural features underpin the improved ion diffusivity and interfacial kinetics discussed in the following section.

### 2.2 Nyquist and DRT Analysis of Ion Transport and Reaction Kinetics

**Figure 3:**
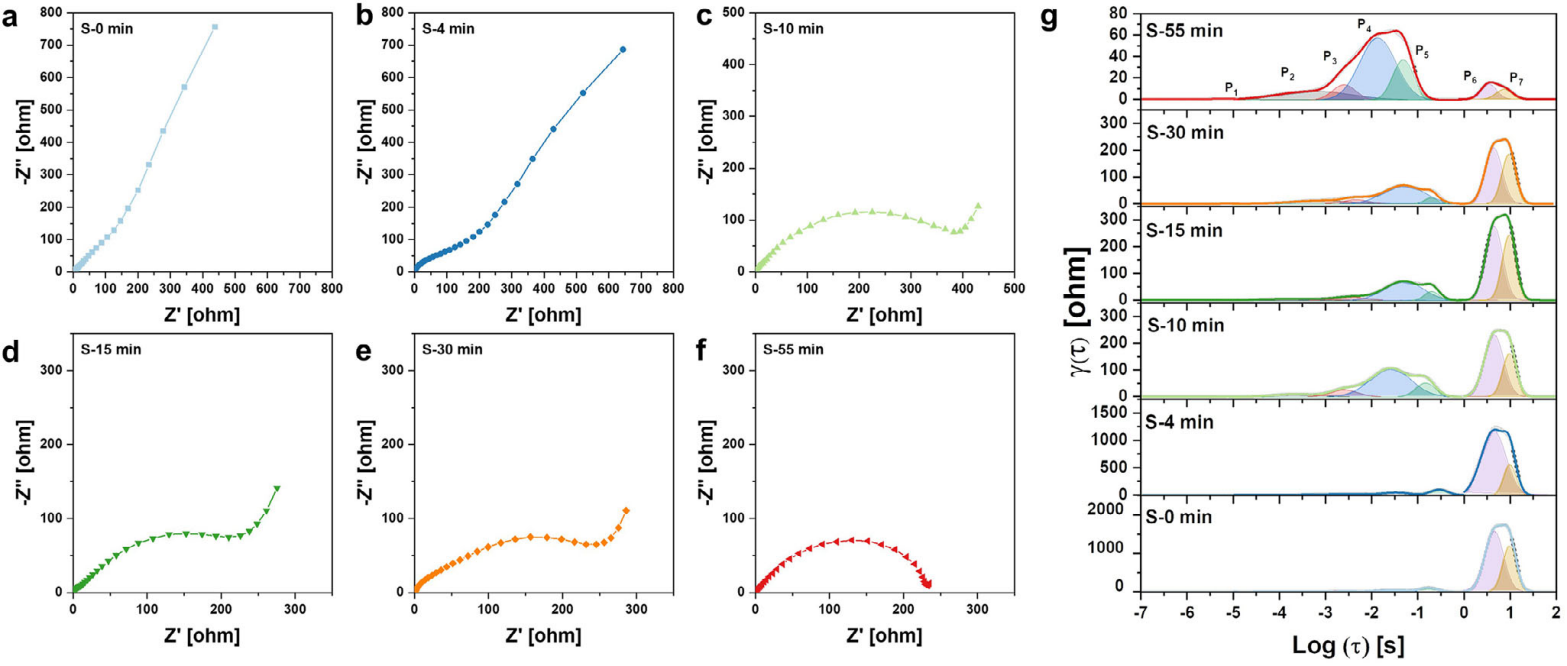
Nyquist and DRT Analysis of Ion Transport and Reaction Kinetics. a–f) Nyquist plots of synthesized at different time, showing a systematic decrease in charge‐transfer resistance and shortened diffusion tails with increasing synthesis time. g) Distributed relaxation time (DRT) spectra derived from the corresponding impedance data. Seven peaks (P_1_–P_7_) are assigned to distinct electrochemical processes: P_1_ – electrolyte resistance, P_2_‐charge transfer at the SEI, P_3_ – anodic charge transfer, P_4_,P_5_ – cathodic charge transfer, P_6_ – interfacial Zn^2+^ diffusion, P_7_ –Zn^2+^ diffusion within cathode.

**Figure** **3** presents Nyquist plots and distributed relaxation time (DRT) analysis to elucidate the impact of synthesis time on ion diffusion and electrochemical reaction kinetics. The low‐frequency tail, associated with Zn^2+^ diffusion resistance, shortens progressively with synthesis time (Figure 3a–f). This improvement stems from the development of a well‐aligned 1D nanofiber architecture, which lowers tortuosity and establishes continuous ion transport pathways, thereby enhancing ionic accessibility and facilitating bulk diffusion. Simultaneously, the charge transfer resistance (*R*_ct_) systematically decreases, reflecting enhanced kinetics at the electrode/electrolyte interphase. This enhancement is attributed to the synergistic effect of K^+^ incorporation and the 1D nanofiber architecture.

The equivalent circuit was represented in Figure S12 (Supporting Information). Each R–CPE (constant phase element) in the equivalent circuit corresponds to a distinct electrochemical process, such as charge transfer at different electrode domains and Zn^2+^ diffusion within electrode. For early‐stage (S‐0 and S‐4 min), the presence of two distinct R–CPE elements in cathodic charge transfer process is attributed to the sluggish kinetics of pristine V_2_O_5_. Specifically, the distribution of electrochemical activity—arising from both surface‐accessible and bulk‐limited Zn^2+^ insertion sites—leads to two separate charge‐transfer processes occurring at different time constants. As the synthesis progresses, the number of R–CPE elements in the equivalent circuit decreases, suggesting that the charge transfer process is improved. Especially, in the S‐55 min, Warburg impedance (W) becomes evident in the fitted circuit, replacing the previously diffusion‐related R‐CPE response. This transition suggests a shift from confined solid‐state diffusion to more continuous, semi‐infinite diffusion behavior—likely due to the improved structural pathways.

This trend is further clarified by DRT analysis, which resolves the dynamic evolution of charge‐transfer and diffusion processes with synthesis time (Figure 3g; Figure S13, Supporting Information).The relaxation time (τ) extracted from DRT represents distinct electrochemical phenomena.^[^ ^42^ ^]^ Peaks with low τ values correspond to electrolyte resistance (P_1_), while those in the mid‐τ region (‐5 < Log (τ) < ‐1) are associated with charge transfer at the SEI (P_2_), anodic (P_3_) and cathodic reactions (P_4_, P_5_). Notably, P_4_ and P_5_ initially appear as two separate peaks, indicating a distinct difference in redox kinetics. However, with increasing synthesis time, these peaks shift toward shorter τ values and eventually merge, signifying accelerated charge transfer process. At Log (τ) > 1, the τ values correspond to Zn^2+^ diffusion. Initially, two distinct peaks are observed, representing Zn^2+^ diffusion at the electrode/electrolyte interphase (P_6_) and Zn^2+^ diffusion within the cathode (P_7_). As synthesis time increases, both P_6_ and P_7_ values decrease significantly, suggesting that the 1D nanofiber facilitates electron/ion diffusion and kinetics.

Therefore, the structural evolution induced by K^+^ intercalation plays a critical role in modulating the cathode's electrochemical behavior by improving Zn^2+^ diffusion pathways and lowering charge‐transfer resistance, suggesting a more homogeneous and kinetically favorable interfacial environment.

### 2.3 Structure‐Driven Enhancement in Rate Capability and Cycling Stability

In AZIBs, dynamic challenges of cathodes—such as sluggish ion diffusion, limited electronic conductivity, and slow conversion reactions—represent key kinetic barriers that limit electrochemical performance.^[^ ^43^ ^]^ These limitations can be mitigated by regulating the interlayer environment in cathode materials, facilitating ion transport.

**Figure 4:**
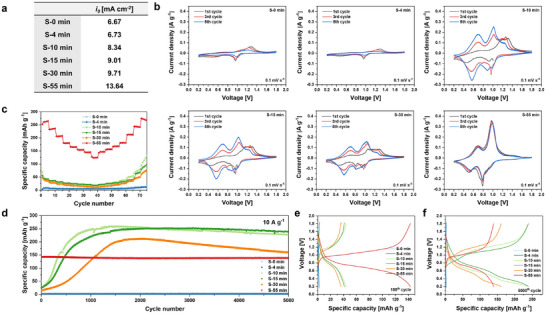
Structure–performance correlation of KVO revealed by electrochemical kinetics and long‐term cycling stability. a) Exchange current density (*i_0_*) values extracted from Tafel analysis as a function of synthesis time. b) Cyclic voltammetry profiles with different synthesis times measured at 0.1 mV s−1. c) Rate performance with 0.5‐1‐2‐4‐6‐8‐10‐15 A g^−1^. d–f) Long‐term cycling performance and charge–discharge profiles of electrodes synthesized at different time points.

To evaluate this, half‐cell Tafel analysis was conducted (**Figure** **4**a; Figure S14, Supporting Information). The exchange current density (*i_0_*), which reflects the charge transfer rate at the electrode/electrolyte interphase, increases systematically from 6.83 to 13.64 mA cm^−2^ as synthesis time progresses.^[^ ^44^, ^45^ ^]^ This enhancement is attributed to the formation of a more accessible electrochemical site enabled by the interlayer expansion and the emergence of 1D nanofiber structures. The K^+^ act as electrostatic buffers (“pillar effect”), alleviating strong Coulombic interactions between Zn^2+^ and the host framework, thereby facilitating faster charge transfer across the interphase.

Cyclic voltammetry (CV) conducted over the initial five cycles (Figure 4b) further corroborates this trend. In early‐stage electrode (S‐0 to S‐4 min), Zn^2+^ diffusion is severely hindered by strong electrostatic interactions. In contrast, partially transformed electrodes (S‐10 to S‐30 min) exhibit three redox pairs, though one pair gradually fades with cycling—indicative of an irreversible structural rearrangement. Meanwhile, the increasing intensity of the remaining redox peaks reflects progressive electrode activation and the formation of electrochemically accessible sites.^[^ ^46^ ^]^ At S‐55 min, distinct redox peaks are maintained across all five cycles, indicating robust reversibility and enhanced structural stability under cycling conditions.

The *b*‐value analysis (Figure S15, Supporting Information), which distinguishes between capacitive and diffusion‐controlled charge storage mechanisms, further supports the kinetic evolution.^[^ ^47^ ^]^ While S‐10 to S‐30 min exhibits predominantly surface‐controlled behavior (*b* ≈ 1), the S‐55 min exhibits a *b*‐value between 0.5 and 1, indicating the coexistence of capacitive and diffusion‐limited processes. This hybrid behavior indicates that Zn^2+^ are involved in both surface interactions and intercalation processes, enhancing ion accessibility throughout the electrode structure.

Figure 4c and Figure S16 (Supporting Information) present the rate capability performance. The S‐0, S‐4 min exhibited extremely poor capacity performance, attributed to strong electrostatic interactions that induce sluggish kinetics and restrict Zn^2+^ diffusion. In contrast, S‐10 to S‐30 min display unexpected capacity increases during retention sequence—a signature of progressive activation. The S‐55 min exhibited outstanding rate capability, delivering a high capacity of 262.79 mAh g^−1^ at 0.5 A g^−1^, and retaining 123.75 mAh g^−1^ even under an ultrafast rate of 15 A g^−1^. The 1D nanofiber network minimizes the complexity of ion diffusion paths, enabling more direct and less resistive Zn^2+^ transport. This straightened ion flux, in turn, mitigates internal concentration polarization and enhances kinetics. Notably, the rate performance of S‐75 min remained nearly identical to that of S‐55 min (Figure S17, Supporting Information), confirming that structural transformation stabilizes by 55 min of synthesis.

Long‐term cycling performance (Figure 4d–f) supports the role of structure in dictating electrochemical stability. At S‐10 to S‐30 min, continuous capacity enhancement was observed for up to 1500 cycles. They are partially transformed and reside in a mixed‐phase regime where V_2_O_5_ coexists with emerging KVO. Consequently, their solid‐state diffusion is improved relative to S‐0 min, and they deliver higher initial capacities than S‐0 min. Upon cycling, these intermediate‐phase electrodes exhibit a gradual capacity increase, reaching over 200 mAh g^−1^, during early cycling because the remaining V_2_O_5_ domains undergo structural rearrangement driven by electrochemical cycling. This activation is further promoted by the insertion of H⁺ and water molecules into the electrode, which expands the interlayer spacing, increases the accessible active sites, and thereby facilitates ion transport. Beyond 1500 cycles, however, structural degradation begins to manifest, likely due to repeated interlayer expansion and collapse induced by the continual insertion/extraction of Zn^2+^ and H^+^. In contrast, the S‐55 min demonstrated exceptional durability, retaining 97.26% of its initial capacity after 5000 cycles at 10 A g^−1^. This remarkable stability is ascribed to the robust 1D nanostructure, which accommodates volume changes and suppresses interlayer collapse under high‐rate cycling.

Furthermore, S‐55 min exhibits durable performance even under low current conditions. Vanadium‐based cathodes often deteriorate at low current because the electrode requires a longer time to approach equilibrium, which in turn increases the likelihood of vanadium dissolution. ^[^ ^48^, ^49^ ^]^ In contrast, S‐55 min retains 83.04% of its initial capacity after 100 cycles at 500 mA g^−1^, underscoring its superior structural stability (Figure S18, Supporting Information).

SEM images before and after cycling further supported to its structure stability (Figure S19, Supporting Information). The S‐55 min retained its original nanofiber morphology with minimal surface change, suggesting superior structural robustness and electrochemical stability. This mechanically compliant and chemically robust framework enables continuous Zn^2+^ transport, mitigates structural degradation, and supports long‐term cycling durability across a wide range of current densities.

Thus, K^+^ intercalation serves as an effective strategy to optimize cathode kinetics by enhancing ion transport, accelerating charge‐transfer processes, and stabilizing the electrode structure.

### 2.4 Quantifying Zn^2+^ Diffusion Revealed by GITT and COMSOL

We quantitatively analyzed Zn^2+^ diffusion behavior through a combination of GITT and COMSOL techniques. Since S‐0 and S‐4 min exhibited extremely poor electrochemical performance, they were excluded from further experiments. The average log (*D_Zn_^2+^*) during the first two cycles were −9.68, −9.59, and −9. 37 cm^2^ s^−1^ for S‐10, S‐15, and S‐30 min, respectively, indicating sluggish Zn^2+^ transport and high polarization (Figure S20, Supporting Information). In contrast, S‐55 min exhibited a significantly higher value of −8.85 cm^2^ s^−1^ (1.41 × 10^−9^ cm^2^ s^−1^), reflecting improved ion diffusion consistent with its superior electrochemical performance.

**Figure 5:**
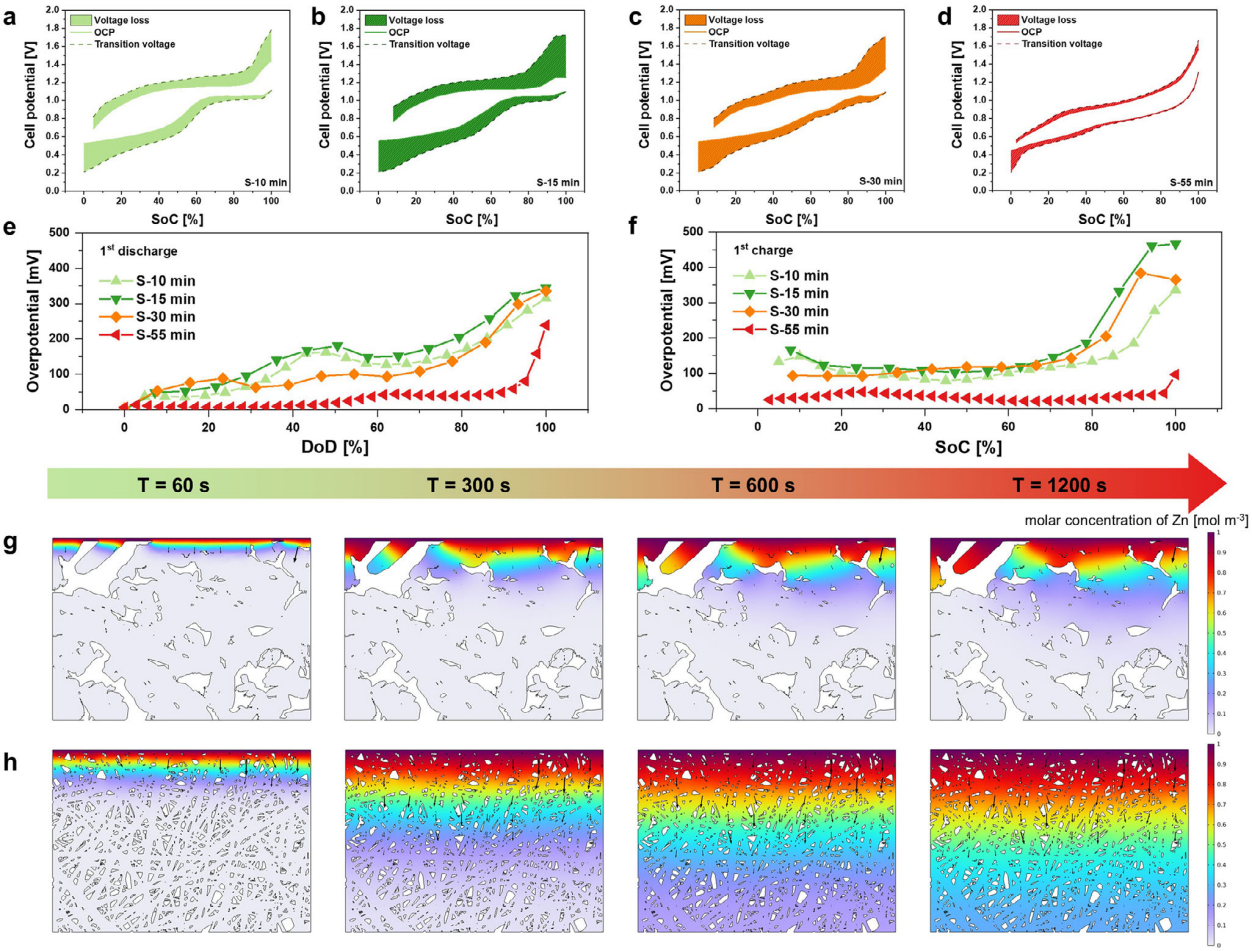
Quantitative Visualization of Zn^2+^ Diffusion behavior via Coupled GITT–Simulation Analysis. a–d) Variation of overpotential (*η*) with SoC. Voltage loss indicated by transient voltage and OCP during charge‐discharge cycling. e,f) Variation of *η* during first discharge (e) and charge (f), highlighting the superior diffusion kinetics of S‐55 min. g,h) COMSOL Multiphysics simulations of Zn^2+^ concentration distribution within the electrode over time (T = 60, 300, 600, and 1200 s) for (g) S‐10 min and (h) S‐55 min.

The overpotential (*η*) was determined using the method presented in Figure S21 (Supporting Information). A higher *η* leads to greater voltage loss, limiting the battery's ability to utilize its capacity. The *η* generally decreases during Zn^2+^ insertion/extraction, a slight increase is observed at deep charge or discharge states, likely due to localized phase transformations or kinetic limitations (**Figure** **5**a–d). The S‐55 min exhibits the lowest *η* across all charge/discharge processes, indicating significantly improved reaction kinetics and Zn^2+^ ion diffusion (Figure 5e,f).

To complement the GITT analysis, finite element simulations were performed using COMSOL Multiphysics to visualize the Zn^2+^ diffusion behavior within the electrode. The electrode geometry was constructed based on SEM images, allowing for the spatial modeling of ion transport under realistic structural constraints. In the S‐10 min, Zn^2+^ diffusion is largely confined to the near‐surface region, with significant concentration gradients and limited penetration into the bulk (Figure 5g). Such tightly packed morphologies restrict Zn^2+^ mobility, resulting in local accumulation at the electrode/electrolyte interphase.

In contrast, the S‐55 min exhibits a more uniform and progressive Zn^2+^ diffusion profile throughout the electrode as time progresses (Figure 5h). This is attributed to the aligned 1D nanofiber morphology, which provides continuous and low‐resistance ion transport pathways into the bulk. The synthesis‐driven COMSOL simulation at multiple time points is shown in Figure S22 (Supporting Information). These simulated results align well with GITT measurements, reinforcing the conclusion that structural evolution—particularly the formation of a low‐tortuosity, aligned architecture—plays a decisive role in enhancing Zn^2+^ transport and mitigating polarization. This simulation result is further corroborated by DRT analysis, where the P_6_ and P_7_—associated with interphase and bulk Zn^2+^ diffusion resistance, respectively—progressively decreases with synthesis time, in line with the improved flux uniformity and ionic accessibility observed in COMSOL.

The dynamic behavior evolves through cycling‐induced structural rearrangement (activation), which progressively opens additional Zn^2+^ insertion pathways. Upon cycling, *η* gradually decreased while *D_Zn_^2+^* increased across all samples (Figures S23 and S24, Supporting Information). After five cycles, the average log (*D_Zn_^2+^*) of S‐10, S‐15, and S‐30 min improved by more than an order of magnitude, and their *η* values approached those of S‐55 min. However, continued Zn^2+^ cycling in these samples can lead to interlayer instability and structural degradation. In contrast, the S‐55 min electrode maintains excellent structural integrity, as reflected by the nearly constant diffusion coefficient before and after five cycles (from 1.41 cm × 10^−9^ to 1.52 × 10^−9^ cm^2^ s^−1^) and consistently low overpotential.

These results indicate that Zn^2+^ diffusion behavior is strongly governed by morphological evolution. Poorly developed early‐stage electrodes exhibit limited ion accessibility and surface‐confined transport, leading to high overpotentials and sluggish diffusion. In contrast, the formation of a low‐tortuosity, 1D‐aligned nanostructure in the fully converted S‐55 min sample promotes homogeneous electron/ion transport, reduces polarization, and supports stable, fast kinetics.

### 2.5 Deciphering Ion Dynamics and Charge Transfer via Operando Electrochemical Analysis

To elucidate the influence of morphological and structural transformations on ion dynamics and charge transfer, *operando*‐EIS analysis was performed (Figure S25, Supporting Information). *Operando*‐EIS enables the simultaneous acquisition of impedance spectra and charge–discharge curves, which is a powerful tool to investigate the impedance evolution as a function of the state of charge (SoC).^[^ ^50^, ^51^, ^52^ ^]^ For a deeper interpretation, the *operando*‐EIS data were converted into the DRT domain, allowing for the separation of charge transfer resistance and mass transport resistance. As illustrated in Figure S26 (Supporting Information), the DRT analysis identified four major relaxation time components, corresponding to distinct resistive contributions:

• τ_1_: Bulk impedance (intrinsic impedance of the electrolyte)
• τ_2_: Charge transfer (associated with charge transfer kinetics at anode/electrolyte interphase and cathode/electrolyte interphase)
• τ_3_: Interfacial diffusion (Zn^2+^ accumulation at the electrode/electrolyte interphase)
• τ_4_: Zn^2+^ diffusion within the cathode

**Figure 6:**
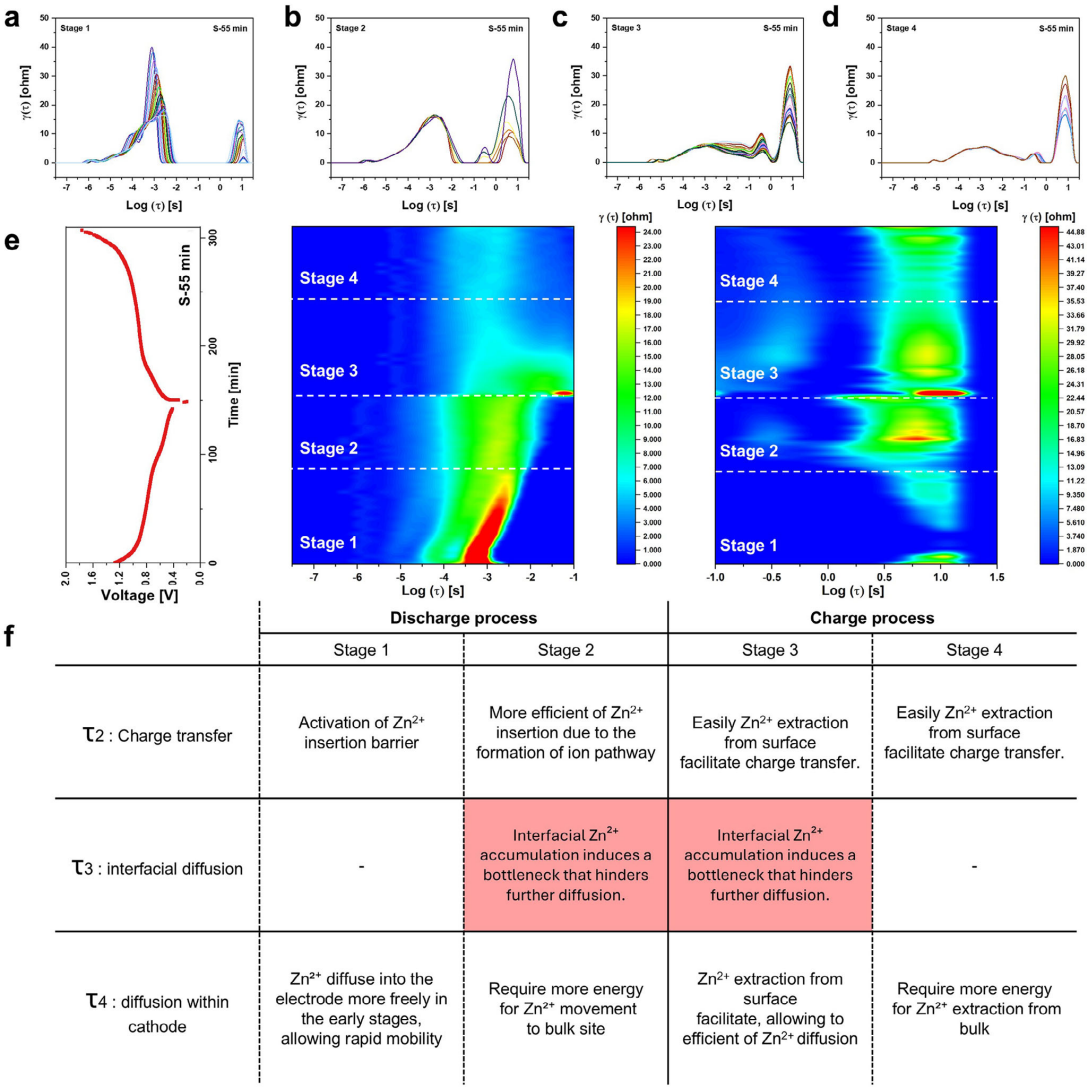
Time‐resolved DRT analysis reveals dynamic Zn^2+^ transport and interphase kinetics during cycling. a–d) Time‐evolved DRT spectra of S‐55 min across four electrochemical stages during discharge (a–b) and charge (c,d). e) Contour plots of corresponding DRT. f) Summary of the change of DRT peaks, and evolution stage 1‐4.

**Figure** **6** presents the *operando*‐DRT analysis of the first cycle for S‐55 min. The discharge–charge process is divided into four stages, where stages 1 and 2 correspond to the discharge process, while stages 3 and 4 represent the charge process. The classification of these stages is based on the dominant influence of charge transfer and mass transfer during the cycling. Figure 6a–d presents the evolution of τ_1_– τ_4_ peaks at different stages of the cycling, while Figure 6e provides a mapped visualization of the overall DRT variations. The overall process is summarized Figure 6f, offering deeper insight into the dynamic and kinetics over the cycling.

Stage 1: Charge Transfer Optimization & Initial Diffusion Pathway Formation

At the early discharge stage (Stage 1), the τ_2_ peak exhibits a gradual decrease in intensity, while the τ shifts toward longer timescales. This trend suggests that the interphase kinetics is progressively delayed, leading to the gradual optimization of charge transfer. A similar trend is also observed in S‐10, S‐15, and S‐30 min, indicating that this phenomenon is an intrinsic characteristic of the first Zn^2+^ insertion process in the cathode (Figure S27, Supporting Information). Additionally, the τ_4_ peak initially decreases, indicating that Zn^2+^ diffusion is relatively facile due to the availability of sufficient ion transport pathways. However, as discharge progresses, the increasing Zn^2+^ concentration within the electrode leads to a subsequent rise in diffusion resistance.

Stage 2: Increased diffusion resistance & Space Charge Effect

In stage 2, a new τ_3_ emerges, whose evolution closely resembles that of τ_4_, while τ_2_ remains unchanged. This observation suggests that τ_3_ originates from diffusion limitations, rather than charge transfer resistance. The τ_3_ signifies Zn^2+^ accumulation at the electrode/electrolyte interphase, which is triggered by hindered Zn^2+^ diffusion within the cathode. As Zn^2+^ ions accumulate at the interface due to limited solid‐state diffusion, the resulting local electric field further restricts ionic mobility across the interphase. Consequently, mass transport becomes the dominant factor governing the reaction kinetics in stage 2.

Stage 3: Onset of Zn^2+^ extraction & Space Charge Effect.

During the charging process, Zn^2+^ migrates along pre‐established diffusion pathways, resulting in relatively smaller impedance variations compared to the discharge process. In stage 3, Zn^2+^ extraction from the electrode surface results in a progressive decrease in τ_2_. τ_3_ initially increases due to the formation of ion accumulation at the interphase, which caused by Zn^2+^ extraction induces a localized charge imbalance. This temporary charge imbalance induces an electric field that restricts Zn^2+^ mobility, further increasing τ_3_. However, as Zn^2+^ extraction from the bulk electrode begins, the interfacial charge imbalance gradually stabilizes leading to a gradual decrease in τ_3_. This τ_3_ emergence clearly marks the onset of interfacial transport bottlenecks, highlighting diffusion‐reaction decoupling as the dominant limiting factor during discharge.

Stage 4: Bulk Zn^2+^ extraction & increased diffusion resistance

In stage 4, τ_3_ progressively decreases, while τ_4_ gradually increases. This trend suggests that Zn^2+^ extraction progresses from the electrode surface (Stage 3) to deeper bulk regions, requiring Zn^2+^ migration from embedded active sites. As a result, diffusion resistance gradually increases Zn^2+^ transport becomes progressively restricted within the electrode structure. Meanwhile, since the charge transfer pathway remains well‐established, the variation in τ_2_ becomes negligible and mass transfer becomes the dominant factor in this stage.

A comparison among the four electrodes (S‐10 to S‐55 min) allows for an in‐depth analysis of their ion diffusion and kinetics using *operando*‐DRT (Figure S27, Supporting Information). Among these, the S‐55 min exhibits the lowest Zn^2+^ diffusion resistance, at both τ_3_ and τ_4_. Notably, τ_3_ is consistently observed in S‐10 to S‐30 min during stage 1, whereas it is absent in S‐55 min. As shown in Figure S28 (Supporting Information), S‐10 min displays early‐stage Zn^2^⁺ accumulation near the electrode surface, which manifests as τ_3_ and leads to the formation of an interfacial bottleneck. In contrast, the S‐55 min electrode shows suppressed τ_3_ and uniform Zn^2^⁺ diffusion, resulting in stable voltage behavior.

To visualize the spatial Zn^2+^ distribution across the electrode/electrolyte interface, cross‐sectional Raman mapping was performed. As shown in Figure S29 (Supporting Information), the Raman scan was conducted along the Y‐direction from the electrode surface toward the separator. Raman mapping was performed at a fixed voltage of 0.7 V, which corresponds to the plateau region of the discharge profile. For the S‐10 min electrode (Figure S29b, Supporting Information), a localized increase in Raman intensity is observed within the first 2 µm near the electrode surface, indicating a non‐uniform Zn^2+^ distribution and interfacial ion accumulation, rather than a gradual concentration gradient. This highly concentrated interfacial region suggests the formation of a space charge layer. In contrast, the S‐55 min electrode (Figure S29c, Supporting Information) exhibits a much more uniform intensity profile across the interface, reflecting efficient Zn^2+^ transport and suppressed interfacial accumulation, consistent with the absence of τ_3_ in *operando‐*DRT analysis.

Furthermore, this electrochemical performance can depend on electrode thickness, as increased mass loading often limits electrolyte infiltration and impedes ion and electron transport within the electrode bulk.^[^ ^53^, ^54^ ^]^ To investigate this, we additionally evaluated a thicker electrode with S‐10 and 55 min. The thicker S‐10 min electrode exhibits a marked change in both charge‐transfer (τ_2_) and diffusion region (τ_4_), as shown in Figure S30 (Supporting Information). This behavior can be attributed to ion migration through the dense electrode structure, which increases the effective ion/electron diffusion path. In contrast, the S‐55 min shows minimal overall resistance variation with increased loading, aside from a slightly increase in τ_4_ associated with Zn^2+^ diffusion (Figure S31, Supporting Information). The well‐aligned 1D nanofiber network and expanded interlayer spacing in S‐55 min facilitate ion transport even at higher loading, thereby minimizing diffusion penalties.

To further investigate how cycling impacts interfacial and bulk transport behavior, we conducted *operando*‐EIS measurements after five cycles. Figure S32 (Supporting Information) presents the *operando*‐EIS profile after five cycles, while its corresponding data were transformed into the DRT domain and displayed in Figure S33 (Supporting Information). The S‐55 min maintains an impedance response similar to its initial state, suggesting that the well‐defined 1D nanostructure stabilizes Zn^2+^ diffusion channels over prolonged cycling. In contrast, the impedance response of S‐10, S‐15, and S‐30 min deviates significantly from their initial state, accompanied by a notable reduction in overall resistance. The dominant improvement in electrochemical performance originates from the suppression of τ_3_ and τ_4_, corresponding to interphase and bulk diffusion resistance, respectively. This suppression is attributed to activation‐induced interlayer expansion and structural reorganization, which collectively optimize Zn^2+^ transport. The observed reduction in diffusion resistance corresponds to an approximately tenfold increase in *D_Zn_^2+^*.

Therefore, we reveal that performance limitations originate from solid‐state Zn^2+^ diffusion within the cathode. The diffusion barriers result in local ion accumulation at the electrode/electrolyte interface, manifested as the τ_3_ component in *operando*‐DRT analysis. Such interfacial ion accumulation is significantly suppressed upon the transition to a 1D nanofiber architecture. These findings underscore that the mitigation of diffusion‐related bottlenecks—rather than enhancement of charge‐transfer kinetics—is the key determinant of long‐life performance in AZIBs.

### 2.6 Revealing Ion Transport Bottlenecks via Coupled Structural–Computational Mapping

**Figure 7:**
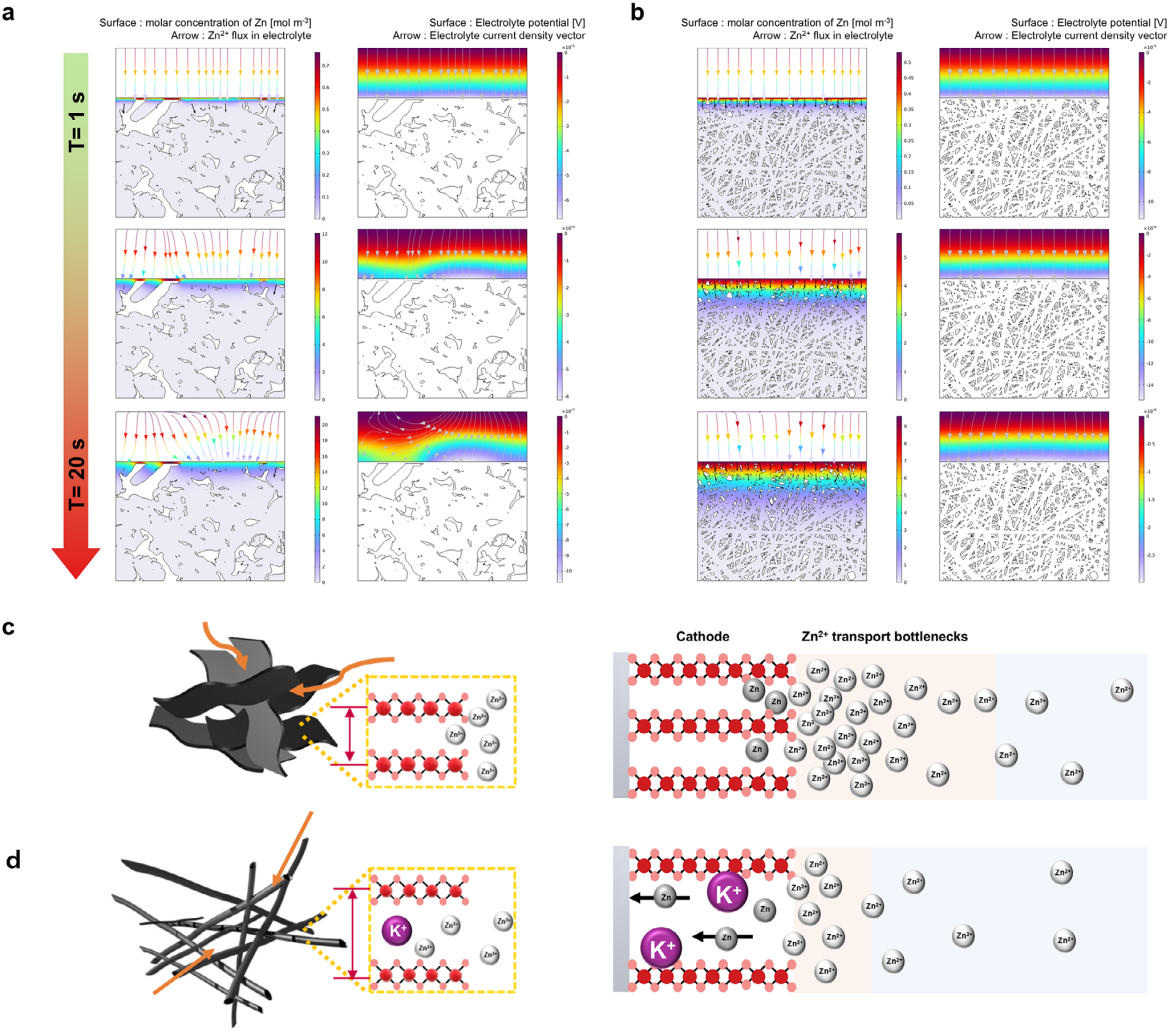
Structural modulation governs Zn^2+^ flux uniformity and electrolyte potential distribution at the cathode/electrolyte interphase. a,b) Time‐dependent evolution of Zn^2+^ concentration, flux vector, and electrolyte potential distribution for a) S‐10 min and b) S‐55 min. c) Schematic illustration of interfacial Zn^2+^ bottlenecks of S‐10 min d) Schematic illustration of S‐55 min.

To further validate the existence of diffusion‐induced transport bottlenecks, finite element simulations were performed using COMSOL Multiphysics. These simulations provide spatially resolved insight into ion accumulation and flux imbalance within the interphase, corroborating the interpretation derived from operando‐DRT analysis. **Figure** **7**a,b depict the time‐dependent evolution of Zn^2+^ flux in the electrolyte, and Zn^2+^ diffusion within the electrode (left), alongside the corresponding electrolyte potential distribution (right). In the S‐10 min electrode, Zn^2+^ flux in the electrolyte becomes progressively distorted due to sluggish Zn^2+^ diffusion within the cathode. As ion transport into the bulk is hindered, Zn^2+^ accumulate near specific regions at the electrode/electrolyte interphase, resulting in spatially non‐uniform flux patterns. The ions are redirected toward areas with relatively higher diffusivity, leading to pronounced curvature in the flux lines. This phenomenon is clearly reflected in the electrolyte potential distribution (right), which exhibits localized bending and potential gradient distortion in response to charge accumulation and nonlinear transport behavior. These findings underscore the emergence of spatially heterogeneous concentration gradients, along with space charge regions, particularly in structurally dense electrodes.

In contrast, the S‐55 min electrode exhibits straight, uninterrupted Zn^2+^ flux lines and a more uniform concentration distribution throughout the electrode depth (Figure 7b). The well‐aligned 1D nanostructure offers a distinct advantage for ion transport by minimizing structural tortuosity and providing continuous, low‐resistance diffusion pathways. This directional alignment enables rapid and uniform ion migration from the electrolyte into the bulk electrode, effectively mitigating interfacial Zn^2+^ congestion. As a result, Zn^2+^ flux is uniformly distributed across the interphase, facilitating consistent electrochemical reactions over the entire electrode surface.

Notably, a slight curvature of the Zn^2+^ flux lines are still observed near the electrode/electrolyte interphase in the S‐55 min at later time points (T > 30 s, Figure S34, Supporting Information). This subtle deviation suggests the presence of a marginal ion accumulation, consistent with the residual τ_3_ peak observed in the operando DRT analysis. Nevertheless, the extent of flux distortion substantially reduced compared to S‐10 min, reinforcing the conclusion that structural alignment significantly alleviates interfacial transport bottlenecks.

Figure 7c,d present schematic illustrations of Zn^2+^ transport near the cathode/electrolyte interphase. Due to a mismatch between the rapid interfacial reaction rate and the sluggish diffusion into the bulk, Zn^2+^ accumulates at the interphase, forming a local space charge region. This phenomenon, driven by interphase kinetics–diffusion mismatch, highlights how structural constraints can lead to ion transport bottleneck.

Taken together, these results establish a direct mechanistic link between electrode architecture, Zn^2+^ diffusion behavior, and interfacial reaction uniformity. The observed distortion of ion flux and electrolyte potential in early‐stage cathodes arises from mismatched diffusion and reaction kinetics, which induces space charge region and potential gradient distortion. In contrast, structurally optimized electrodes suppress these effects by facilitating continuous ion transport into the bulk. These findings not only rationalize the synthesis‐dependent performance disparities but also underscore the critical role of electrode architecture in governing charge transport and reversibility in aqueous Zn‐ion batteries.

## 3 Conclusion

This work provides insights into how synthesis time modulates the structural and morphological evolution of K_2_V_6_O_16_·1.5H_2_O cathodes and its subsequent impact on Zn^2+^ ion diffusion and charge transfer kinetics in AZIBs. To identify the major contributors to overpotential in AZIBs, we employed *operando*‐EIS and DRT analysis, enabling the decoupling of charge transfer resistance and ion diffusion. Our methodology elucidated how these processes evolve during cycling and their role in electrochemical polarization. These operando analyses reveal that limited Zn^2^⁺ diffusion within the cathode leads to interfacial ion accumulation, which in turn induces a transport bottleneck that restricts ion mobility. To validate and visualize this behavior, we implemented finite element simulations using COMSOL Multiphysics. These simulations confirmed that structurally dense cathodes exhibit distorted Zn^2+^ flux lines, localized electric potential gradients, and accumulation‐driven space charge region—corroborating the DRT‐based interpretation. The optimized K_2_V_6_O_16_·1.5H_2_O cathode, synthesized under controlled conditions, exhibited a 1D nanofiber morphology with expanded interlayer spacing, effectively minimizing diffusion barriers and facilitating charge transport. Consequently, it demonstrated enhanced rate capability, lower polarization, and superior long‐term cycling stability. These findings establish a direct structure‐performance correlation, providing fundamental insights into electrochemical reaction kinetics in AZIB cathodes. The comprehensive understanding developed in this work serves as a design framework for next‐generation Zn‐ion battery cathodes, bridging the gap between materials engineering and electrochemical kinetics to advance high‐performance, durable energy storage systems.

## 4 Experimental Section

### Synthesis of the Potassium Vanadate (KVO)

Vanadium pentoxide (V_2_O_5_, 99%, Sigma–Aldrich, 2.2 mmol) and potassium persulfate (K_2_S_2_O_8_, 99%, Sigma–Aldrich, 0.88 mmol) were dissolved in 100 mL of deionized (DI) water under stirring, forming a yellow solution. Sonication was performed in cycles of 2 s on and 1 s off for a total duration of 55 min at an amplitude of 80% via ultrasonic transducer (Sonics & Materials Inc., USA, VCX 750, 20 kHz). Through sonochemical reaction, the structure and morphology dynamically evolved in response to the reaction environment. After 55 min, the solution color transitioned from yellow to dark red, indicating the completion of potassium vanadate formation. The resulting precipitate was collected via filtration, washed thoroughly with DI water, and dried in a vacuum oven at 60 °C for 12 h, yielding the potassium vanadate material.

To elucidate the influence of time‐dependent morphological and structural evolution on electrochemical performance, samples were systematically extracted at distinct synthesis stages: the initial stage (0–4 min), the intermediate stage (10–30 min), and the final stage (55 min). These points were selected to capture critical transitions in ion dynamics and interphase kinetics during material formation. It was denoted samples synthesized with sonification for X minutes as S‐X min (where X represents the synthesis time).

### Material Characterization

The morphology and structural characteristics of the synthesized materials were examined using scanning electron microscopy (SEM, SU8220, Hitachi) equipped with an energy dispersive X‐ray spectrometer (EDS) and transmission electron microscopy (TEM, JXA8530F, JEOL). The crystallinity and phase composition were analyzed by X‐ray diffraction (XRD, EMPYREAN, Panalytical) using CuKα_1_ radiation (λ = 0.15 406 nm). To investigate the chemical bonding and molecular structure of the S‐X min, Fourier transform infrared spectroscopy (FT‐IR, Frontier, PerkinElmer) and Raman spectroscopy (MantaRay, WEVE) were performed. The chemical states of the vanadate nanofibers and the presence of oxygen vacancies were further identified using X‐ray photoelectron spectroscopy (XPS, NEXSA, Thermo Fisher) and electron spin resonance spectroscopy (ESR, EMXplus‐9.5/2.7, Bruker). Thermal stability and decomposition behavior were explored using thermogravimetric analysis (TGA, Q500, TA Instruments) after 24 h vacuum drying of synthesis materials. Moreover, the specific surface area and porosity of the synthesized materials were determined via N_2_ adsorption‐desorption analysis at 77 K using liquid nitrogen. The concentration of dissolved vanadium ions in the electrolyte after cathode immersion was quantified using inductively coupled plasma mass spectrometry (ICP‐MS, NexIon 2000, PerkinElmer). Additionally, the change in electrolyte pH after cathode immersion was measured using a pH meter (Orion Star A, Thermo Fisher).

### Cell Fabrication

The cathode was fabricated using active material, carbon black (Super P, TIMCAL), and poly(vinylidene difluoride) (PVDF, HSV 900, Kynar) in a mass ratio of 7:2:1. To form a homogeneous slurry, the powders were dispersed in N‐methyl‐2‐pyrrolidone (NMP, Sigma–Aldrich) and mixed thoroughly. The resulting slurry was then uniformly coated onto carbon paper, achieving a mass loading of 1–2 mg cm^−2^. The aqueous electrolyte was prepared by dissolving 2.58 M zinc trifluoromethanesulfonate (Zn(OTF) _2_, Alfa‐Assar, 98%) in deionized water. A glass fiber separator (GF/C, Whatman) was used, and 250 µL of the prepared electrolyte was applied to the separator.

### Electrochemical Measurement

All electrochemical measurements were performed in a temperature‐controlled battery chamber. Electrochemical impedance spectroscopy (EIS) was conducted using a Bio‐Logic SP‐200 over a frequency range of 0.1 Hz to 1 MHz. Cyclic voltammetry (CV) was carried out with an increasing scan rate every 5 cycles to evaluate the electrochemical kinetics. Galvanostatic intermittent titration technique (GITT) was employed to analyze the overpotential and Zn^2+^ diffusion coefficient (*D_Zn_^2+^*) by applying galvanostatic charge–discharge at 50 mA g^−1^, followed by a 10 min relaxation step, within a voltage range of 1.8 to 0.2 V (Neware, China). The *D_Zn_^2+^* was calculated by equation 1.

(1) $$D_{Zn^{2+}}=\frac{4L^{2}}{\pi \tau }{\left(\frac{\Delta E_{s}}{\Delta E_{t}}\right)}^{2}$$

where L is the thickness of the electrode (cm), which corresponds to the ion diffusion length. τ is the relaxation time (s), and △𝐸_s_ refers to the steady‐state potential change (V) induced by the current pulse. △𝐸_t_ indicates the potential change (V) observed during a constant current pulse after compensating for the iR drop.^[^ ^55^, ^56^ ^]^ Cycling performance was assessed at 10 A g^−1^ after pre‐cycling at 0.5, 1, 2, 4, 6, and 8 mA g^−1^ for 3 cycles. Tafel analysis of the half‐cell was conducted within a potential window of −0.25 to 0.25 V versus open circuit voltage (OCV) at a scan rate of 1 mV s^−1^.

### COMSOL Simulation

COMSOL Multiphysics 6.2 was employed to simulate the Zn^2+^ transport and electrochemical reaction at the cathode/electrolyte interphase. The simulation geometry was reconstructed from SEM images to reflect realistic porous electrode structures and the diffusion coefficient of Zn^2+^ was derived from the results of GITT. A coupled model incorporating the Tertiary Current Distribution (Nernst–Planck, TCD) and Transport of Diluted Species (TDS) physics was used to evaluate the interplay between ion transport, electrochemical reaction, and solid diffusion. At the cathode/electrolyte interphase, Zn^2+^ reduced via the reaction:

(2) $$Zn^{2+}+2e\;\to Zn$$

and the resulting Zn was modeled as a diffusing species within the electrode matrix using the TDS module. The local generation rate of Zn was computed based on the Zn^2^⁺ flux derived from the TCD module and introduced as a source term in the TDS module.

(3) $$j=j_{0}\cdot \exp\;\left(-\alpha \cdot C_{Zn}\right)$$

where j_0_ was derived from experimental Tafel analysis and used as an initial input parameter. The reaction rate constant was modulated by a suppression factor α, set to 0.5, to reflect the moderate inhibition effect of Zn generation on interfacial reaction kinetics. Through this TCD–TDS coupling, the model enables spatial resolution of the variation in reaction kinetics and electrolyte potential distribution.

### Operando‐Raman Measurement

The electrochemical Raman measurements were carried out on an in situ confocal microscope Raman (MantaRay, WEVE) and vertical optical cell (SB2300, EL frontier). A 785 nm laser with 1800 grating, 60× microscope objective was used in all measurements. Raman frequency was calibrated by a Si wafer during each experiment. Raman mapping was conducted after discharging to 0.7 V, during which the CV voltage was held constant. The stage was moved along the Y‐axis in 0.1 µm increments from the electrode surface toward the separator to acquire spatially resolved spectra.

### Operando‐EIS Measurement

The in situ electrochemical impedance spectroscopy (EIS) measurements were performed within a voltage window of 0.2–1.8 V (SP‐200, Bio‐Logic). Z‐3D‐analysis software (TOYO Corporation, Japan) was applied to obtain the instantaneous impedance at a certain time from a 3D Nyquist diagram composed of a real axis, an imaginary axis, and a time axis. Impedance spectra were recorded over a frequency range of 1 MHz to 0.1 Hz with applied AC amplitude was 5 mV_rms_, while the DC current density was maintained at 100 mA g^−1^. To analyze each resistance component in *operando*‐EIS, an equivalent circuit fitting was performed using ZView (Scribner Associates, Inc.) software. The corresponding *operando*‐distributed relaxation time (DRT) analysis was conducted using a MATLAB‐based peakfit script to extract relaxation processes from the impedance data.^[^ ^57^ ^]^

## Conflict of Interest

The authors declare no conflict of interest.

## Supporting information



Supporting Information

## Data Availability

The data that support the findings of this study are available from the corresponding author upon reasonable request.

## References

[1] H. Fan , K. Liu , X. Zhang , Y. Di , P. Liu , J. Li , B. Hu , H. Li , M. Ravivarma , J. Song , eScience 2024, 4, 100202.

[2] L. Kou , Y. Wang , J. Song , T. Ai , W. Li , P. Wattanapaphawong , K. Kajiyoshi , M. Y. Ghotbi , Y. Feng , Inorg. Chem. Front. 2024, 11, 1949.

[3] J. Wei , P. Zhang , J. Sun , Y. Liu , F. Li , H. Xu , R. Ye , Z. Tie , L. Sun , Z. Jin , Chem. Soc. Rev. 2024, 53, 10335. 39253782 10.1039/d4cs00584h

[4] B. T. Heligman , K. J. Kreder , A. Manthiram , Joule 2019, 3, 1051.

[5] L. E. Blanc , D. Kundu , L. F. Nazar , Joule 2020, 4, 771.

[6] J. Shin , J. Lee , Y. Park , J. W. Choi , Chem. Sci. 2020, 11, 2028. 32180925 10.1039/d0sc00022a PMC7053421

[7] L. Li , S. Jia , Y. Shi , C. Wang , H. Qiu , Y. Ji , M. Cao , D. Zhang , Inorg. Chem. Front. 2024, 11, 4485.

[8] S. D. Pu , B. Hu , Z. Li , Y. Yuan , C. Gong , Z. Ning , C. Chau , S. Yang , S. Zhang , L. Pi , Y. T. Tang , J. Yue , T. J. Marrow , X. Gao , P. G. Bruce , A. W. Robertson , Joule 2023, 7, 366.

[9] Z. Xing , Y. Sun , X. Xie , Y. Tang , G. Xu , J. Han , B. Lu , S. Liang , G. Chen , J. Zhou , Angew. Chem. ‐ Int. Ed. 2023, 62, 202215324. 10.1002/anie.202215324 36446732

[10] Y. Song , P. Ruan , C. Mao , Y. Chang , L. Wang , L. Dai , P. Zhou , B. Lu , J. Zhou , Z. He , Nanomicro Lett 2022, 14, 218. 36352159 10.1007/s40820-022-00960-z PMC9646683

[11] L. Zhang , D. Fang , F. Wang , J. Yi , M. Wang , T. Hu , Y. Zhao , Chem. Eng. J. 2025, 506, 159920.

[12] D. Zhang , W. Wang , S. Li , X. Shen , H. Xu , Energy Storage Mater. 2024, 69, 103436.

[13] J. Huang , Z. Wang , M. Hou , X. Dong , Y. Liu , Y. Wang , Y. Xia , Nat. Commun. 2018, 9, 2906. 30046036 10.1038/s41467-018-04949-4 PMC6060179

[14] L. Li , Y. Zhang , J. Zhou , W. Huang , C. Liu , H. Zhu , Y. Zheng , Z. Wang , J. Mater. Sci. Mater. Electron. 2025, 36, 97.

[15] Y. Zeng , X. F. Lu , S. L. Zhang , D. Luan , S. Li , X. W. Lou , Angew. Chem. ‐ Int. Ed. 2021, 60, 22189. 10.1002/anie.202107697 PMC8518934 34313363

[16] G. Li , W. Yu , Q. Diao , Y. Zhang , F. Tang , X. Luo , L. Yan , X. Zhao , G. Li , ChemPhysChem 2024. 10.1002/cphc.202400860 39470129

[17] X. Chen , H. Zhang , J. H. Liu , Y. Gao , X. Cao , C. Zhan , Y. Wang , S. Wang , S. L. Chou , S. X. Dou , D. Cao , Energy Storage Mater. 2022, 50, 21.

[18] S. Liu , L. Kang , J. M. Kim , Y. T. Chun , J. Zhang , S. C. Jun , Adv. Energy Mater. 2020, 10, 2000477.

[19] T. Lv , Y. Peng , G. Zhang , S. Jiang , Z. Yang , S. Yang , H. Pang , Adv. Sci. 2023, 10, 2206907. 10.1002/advs.202206907 PMC10131888 36683227

[20] H. Liu , X. Hou , Q. Zhang , W. Peng , Y. Li , X. Fan , Adv. Energy Mater. 2025, 15, 2406171.

[21] B. Sambandam , V. Soundharrajan , S. Kim , M. H. Alfaruqi , J. Jo , S. Kim , V. Mathew , Y. K. Sun , J. Kim , J. Mater. Chem. A Mater. 2018, 6, 15530.

[22] Y. So , H. Seo , S. H. Lee , E. Lee , J. Lee , J. Kang , Y. Y. Kim , B.‐H. Kim , S. Mhin , J. Mater. Chem. A Mater. 2025, 13, 8761.

[23] S. Li , X. Xu , W. Chen , J. Zhao , K. Wang , J. Shen , X. Chen , X. Lu , X. Jiao , Y. Liu , Y. Bai , Energy Storage Mater. 2024, 65, 103175.

[24] P. Falun , L. Ngamwongwan , S. Singsen , M. Chotsawat , P. Komen , A. Junkaew , S. Suthirakun , J. Phys. Chem. C 2024, 128, 10774.

[25] R. Li , H. Zhang , Q. Zheng , X. Li , J. Mater. Chem. A Mater. 2020, 8, 5186.

[26] T. Lv , Y. Peng , G. Zhang , S. Jiang , Z. Yang , S. Yang , H. Pang , Adv. Sci. 2023, 10, 2206907. 10.1002/advs.202206907 PMC10131888 36683227

[27] Y. Ding , L. Zhang , X. Wang , L. Han , W. Zhang , C. Guo , Chin. Chem. Lett. 2023, 34, 107399.

[28] J. Ding , H. Gao , D. Ji , K. Zhao , S. Wang , F. Cheng , J. Mater. Chem. A 2021, 9, 5258.

[29] C. Liu , Z. Neale , J. Zheng , X. Jia , J. Huang , M. Yan , M. Tian , M. Wang , J. Yang , G. Cao , Energy Environ. Sci. 2019, 12, 2273.

[30] C. Julien , G. A. Nazri , O. Bergström , Phys. Status Solidi B Basic Res. 1997, 201, 319.

[31] D. Batyrbekuly , B. Laïk , J. P. Pereira‐Ramos , Z. Bakenov , R. Baddour‐Hadjean , J. Energy Chem. 2021, 61, 459. 10.1002/cssc.201903072 31799803

[32] P. Chithaiah , G. T. Chandrappa , J. Livage , Inorg. Chem. 2012, 51, 2241. 22313349 10.1021/ic202260w

[33] G. T. Chandrappa , P. Chithaiah , S. Ashoka , J. Livage , Inorg. Chem. 2011, 50, 7421. 21774460 10.1021/ic2005858

[34] S. H. Lee , J. Han , O. S. Jeon , Y. Park , D. Hong , A. Mirzaei , J. Kim , M. K. Shin , Y. J. Yoo , M. S. Choi , J. Yoo , S. Y. Park , Compos. B Eng. 2024, 275, 112456.

[35] Y. Zhang , H. Xu , Y. He , H. Bian , R. Jiang , Q. Zhao , D. Li , A. Wang , D. Sun , Solid State Ion 2024, 416, 116658.

[36] Y. Li , W. Yang , W. Yang , Y. Huang , G. Wang , C. Xu , F. Kang , L. Dong , J. Energy Chem. 2021, 60, 233.

[37] B. Sambandam , V. Soundharrajan , S. Kim , M. H. Alfaruqi , J. Jo , S. Kim , V. Mathew , Y. K. Sun , J. Kim , J. Mater. Chem. A Mater. 2018, 6, 15530.

[38] R. Lindström , V. Maurice , S. Zanna , L. Klein , H. Groult , L. Perrigaud , C. Cohen , P. Marcus , Surf. Interface Anal. 2006, 38, 6.

[39] G. A. Sawatzky , D. Post , Phys. Rev. B 1979, 20, 1546.

[40] N. Lakshminarasimhan , J. Li , H. C. Hsu , M. A. Subramanian , J. Solid State Chem. 2022, 312, 123279.

[41] S. Kumar , F. Maury , N. Bahlawane , Mater. Today Phys. 2017, 2, 1.

[42] Y. Lu , C. Z. Zhao , J. Q. Huang , Q. Zhang , Joule 2022, 6, 1172.

[43] Y. Zhao , Y. Wang , J. Li , J. Xiong , Q. Li , K. K. Abdalla , Y. Zhao , Z. Cai , X. Sun , eScience 2025, 5, 100331.

[44] Y. H. Fang , Z. P. Liu , ACS Catal. 2014, 4, 4364.

[45] S. Singha Roy , R. Madhu , A. Karmakar , S. Kundu , ACS Mater. Lett. 2024, 6.

[46] P. Jing , W. Wei , W. Luo , X. Li , F. Xu , H. Li , M. Wei , D. Yu , Q. Zhu , G. Liu , Inorg. Chem. Commun. 2020, 117, 107953.

[47] N. K. Mishra , R. Mondal , T. Maiyalagan , P. Singh , ACS Omega 2022, 7, 1975. 35071886 10.1021/acsomega.1c05356 PMC8771951

[48] X. Dou , X. Xie , S. Liang , G. Fang , Sci. Bull. 2024, 69, 833. 10.1016/j.scib.2024.01.029 38302333

[49] S. Liu , J. He , D. sheng Liu , M. Ye , Y. Zhang , Y. Qin , C. C. Li , Energy Storage Mater. 2022, 49, 93.

[50] H. Watanabe , S. Omoto , Y. Hoshi , I. Shitanda , M. Itagaki , J. Power Sources 2021, 507, 230258.

[51] M. Itagaki , K. Honda , Y. Hoshi , I. Shitanda , J. Electroanal. Chem. 2015, 737, 78.

[52] M. Itagaki , N. Kobari , S. Yotsuda , K. Watanabe , S. Kinoshita , M. Ue , J. Power Sources 2004, 135, 255.

[53] Y. X. Song , Z. Y. Zhong , M. J. Chen , Y. Q. Ding , M. Zhou , Z. X. Liu , S. Q. Liang , G. Z. Fang , J. Cent. South Univ. 2024, 31, 4536.

[54] Y. Song , M. Chen , Z. Zhong , Z. Liu , S. Liang , G. Fang , Nat. Commun. 2025, 16, 787. 40169564 10.1038/s41467-025-58153-2 PMC11961598

[55] M. Jia , W. Zhang , X. Cai , X. Zhan , L. Hou , C. Yuan , Z. Guo , J. Power Sources 2022, 543, 231843.

[56] Z. Wu , C. Lu , Y. Wang , L. Zhang , L. Jiang , W. Tian , C. Cai , Q. Gu , Z. Sun , L. Hu , Small 2020, 16, 2000698. 10.1002/smll.202000698 32776405

[57] T. H. Wan , M. Saccoccio , C. Chen , F. Ciucci , Electrochim. Acta 2015, 184, 483.

