# Supplementary material for: Aligned Nanostructures Resolve Zn2+ Transport Bottlenecks via Interfacial Kinetics–Diffusion Coupling in Aqueous Zinc‐Ion Batteries
Source: Adv Sci (Weinh). 2025 Nov 20;13(7):e12691. doi: 10.1002/advs.202512691 (PMC12866781; doi:10.1002/advs.202512691)
Supplement: Supplementary file 1 — Supporting Information [file ADVS-13-e12691-s001.docx]

Supporting Information

**Aligned Nanostructures Resolve Zn^2+^ Transport Bottlenecks via Interfacial Kinetics–Diffusion Coupling in Aqueous Zinc-Ion Batteries**

*Juyeon Han^1^, Eunha Seo^1^, Subeen Park^1^, Se Hun Lee^2,*^ and Jeeyoung Yoo^1,*^*

**
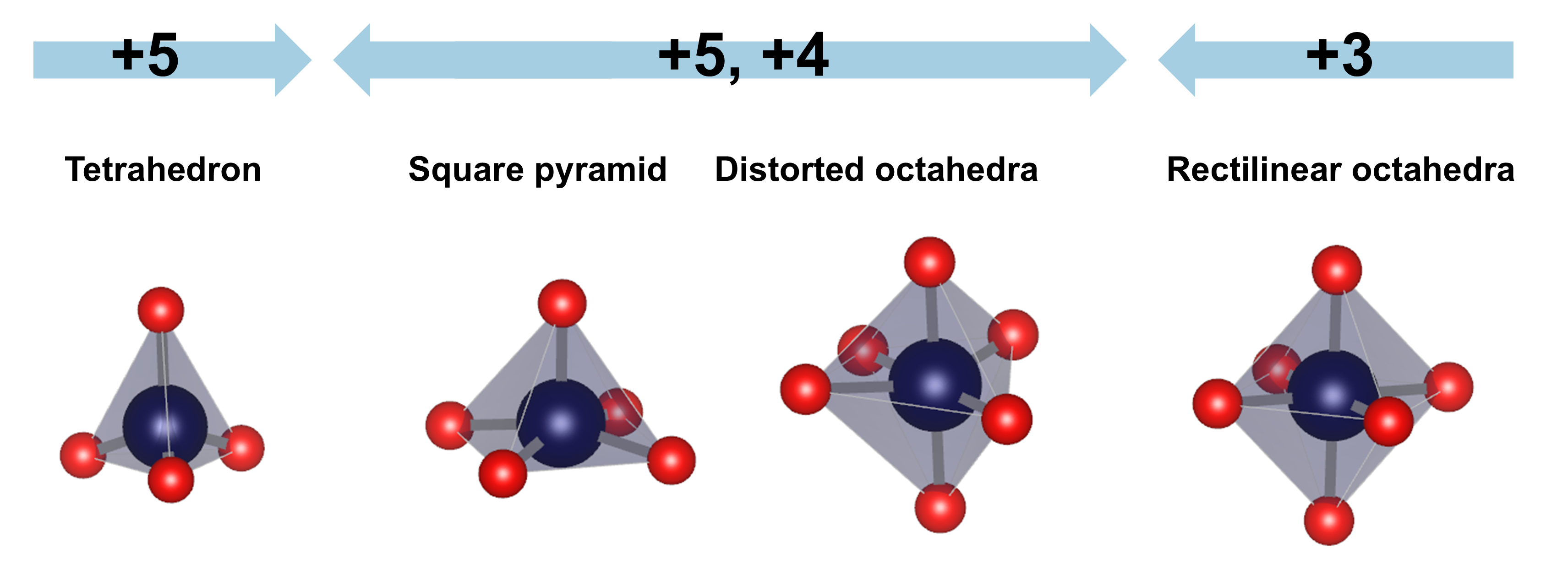
**

**Figure S1.** Diverse coordination environments in vanadium oxide: Polyhedral configurations of V^n+^ in various structural lattices.


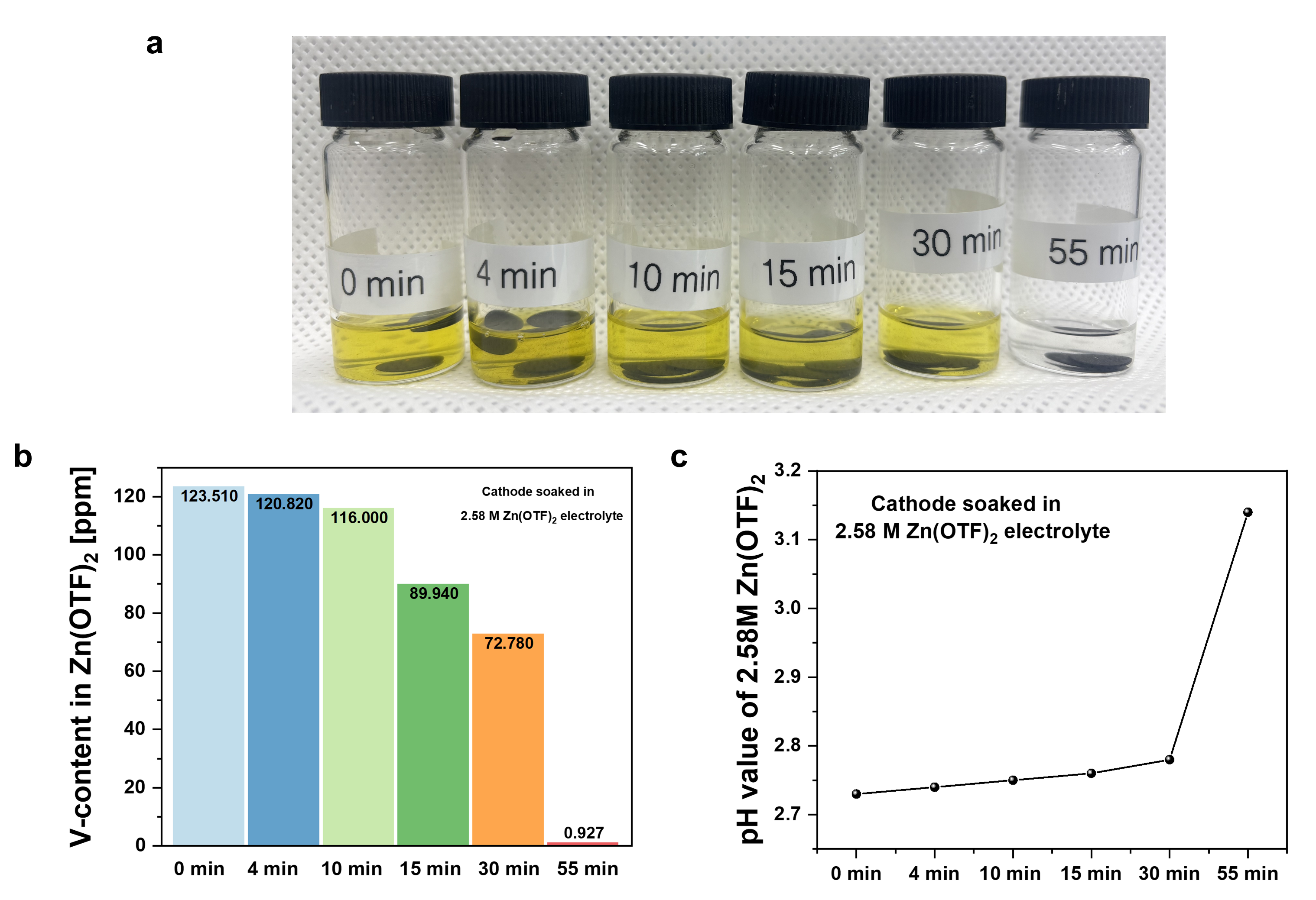


**Figure S2.** Time-dependent V content and pH in the electrolyte after electrode soaking for 1-month. a) Visual color change of electrolyte with cathode synthesized at different durations (0 to 55 min). b) V content in the electrolyte (ppm) measured by ICP. c) Corresponding pH changes of the electrolyte.

**
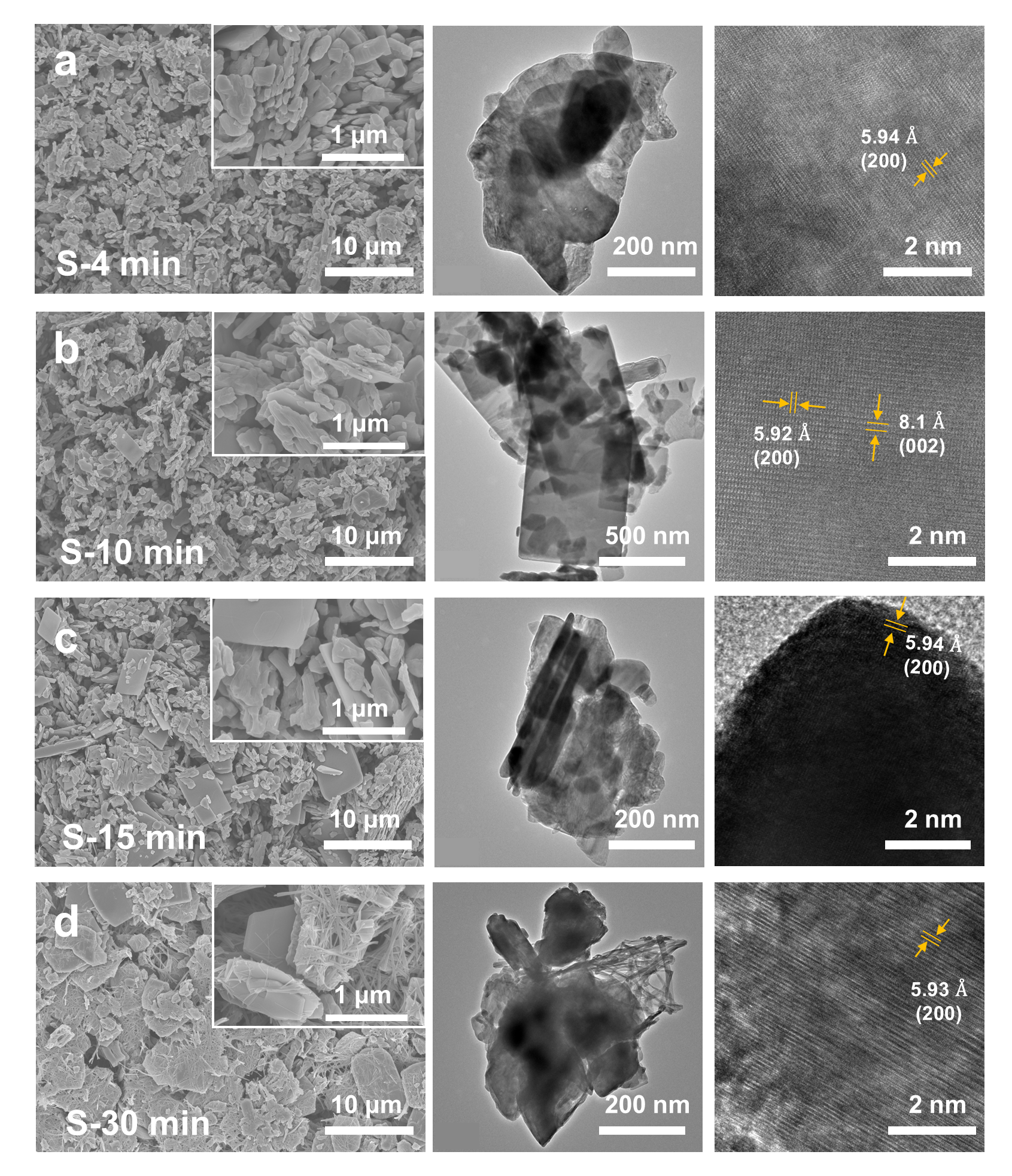
**

**Figure S3.** Morphological and structural evolution of vanadate nanofibers based on SEM and TEM images. a) S-4 min. b) S-10 min. c) S-15 min. d) S-30 min.

**
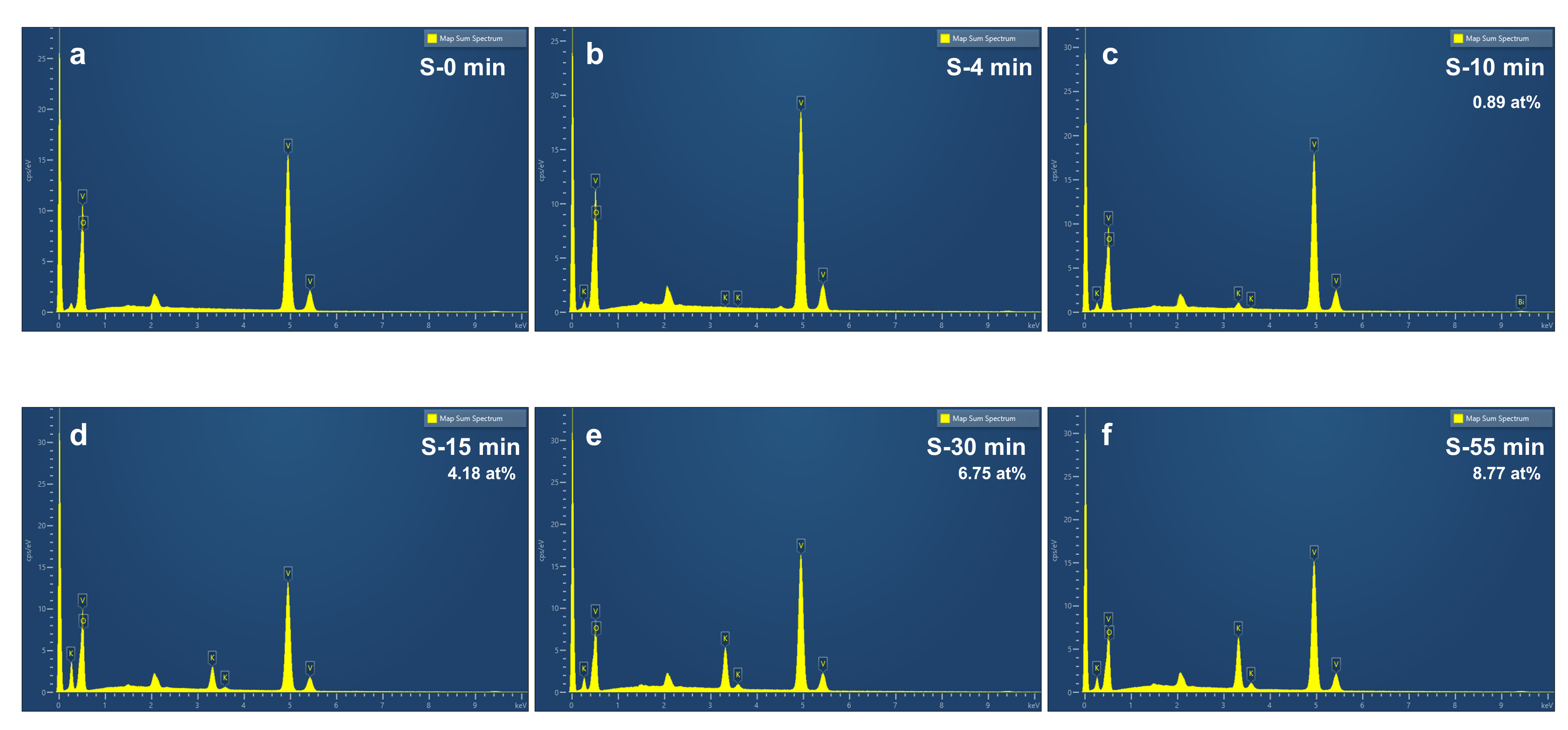
**

**Figure S4.** Elemental mapping analysis of V, O, and K during synthesis. a) S-0 min. b) S-4 min. c) S-10 min. d) S-15 min. e) S-30 min. f) S-55 min. K^+^ is first detected at S-10 min, confirming the onset of potassium intercalation into the vanadate.


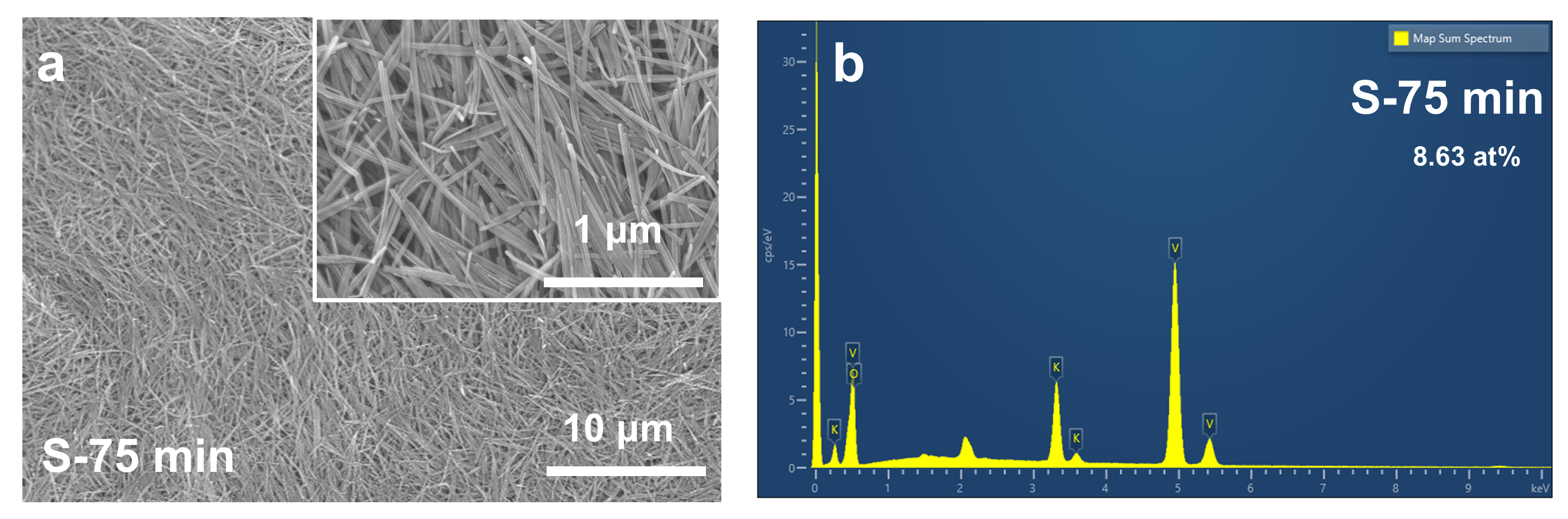


**Figure S5.** EDS analysis of V, O, and K of S-75 min. S-75 min shows no further morphological or compositional changes, indicating completion of the transformation at S-55 min.


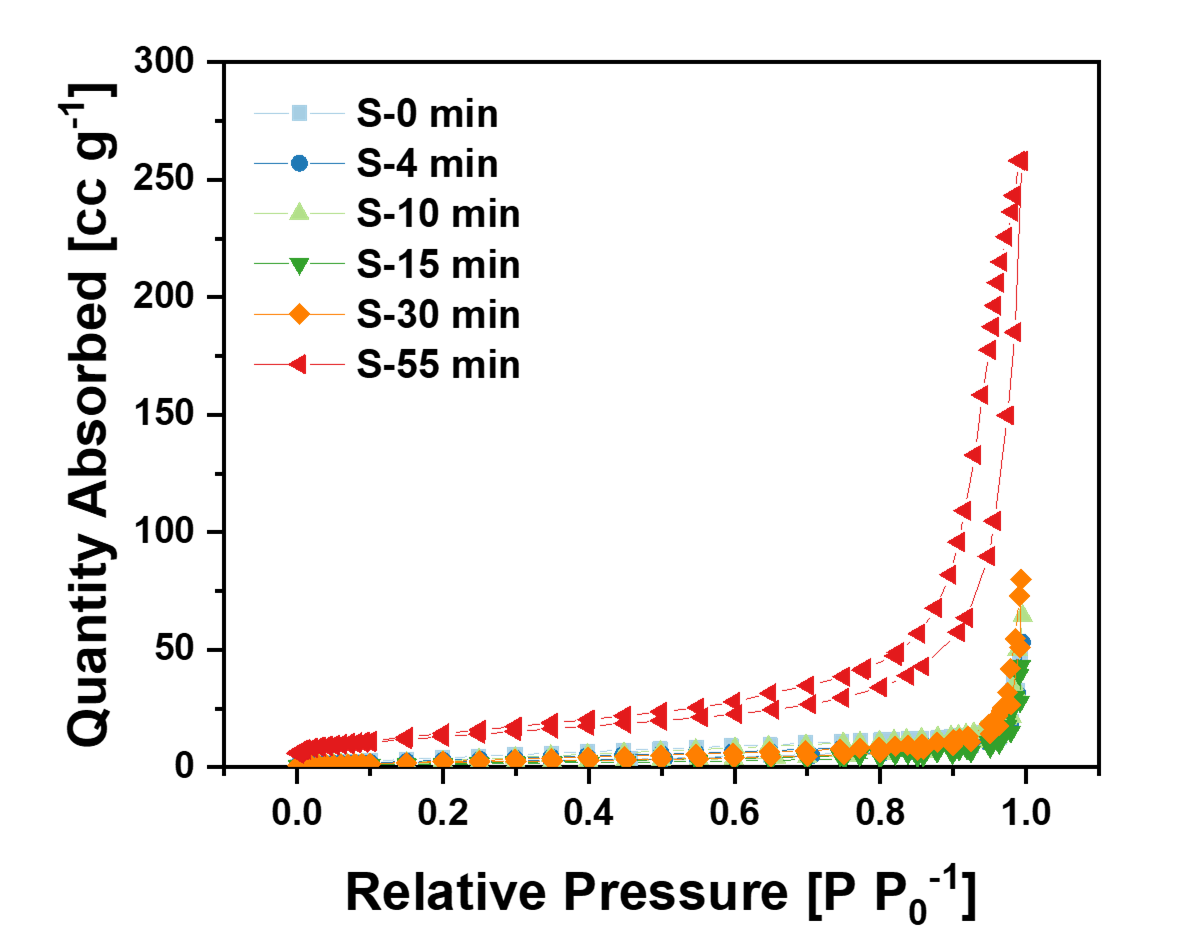


**Figure S6.** N_2_ adsorption–desorption isotherms as a function of synthesis time.

|  | **S-0 min** | **S-4 min** | **S-10 min** | **S-15 min** | **S-30 min** | **S-55 min** |
| --- | --- | --- | --- | --- | --- | --- |
| **Surface area**  **[m^2^ g^−1^]** | 7.406 | 8.524 | 7.662 | 7.862 | 7.986 | 48.254 |
| **Pore volume**  **[CC g^−1^]** | 0.072 | 0.081 | 0.098 | 0.112 | 0.123 | 0.395 |
| **Pore diameter**  **[nm]** | 3.402 | 3.391 | 3.401 | 3.403 | 3.404 | 43.007 |

**Table 1.** Corresponding detailed values of Figure S6.


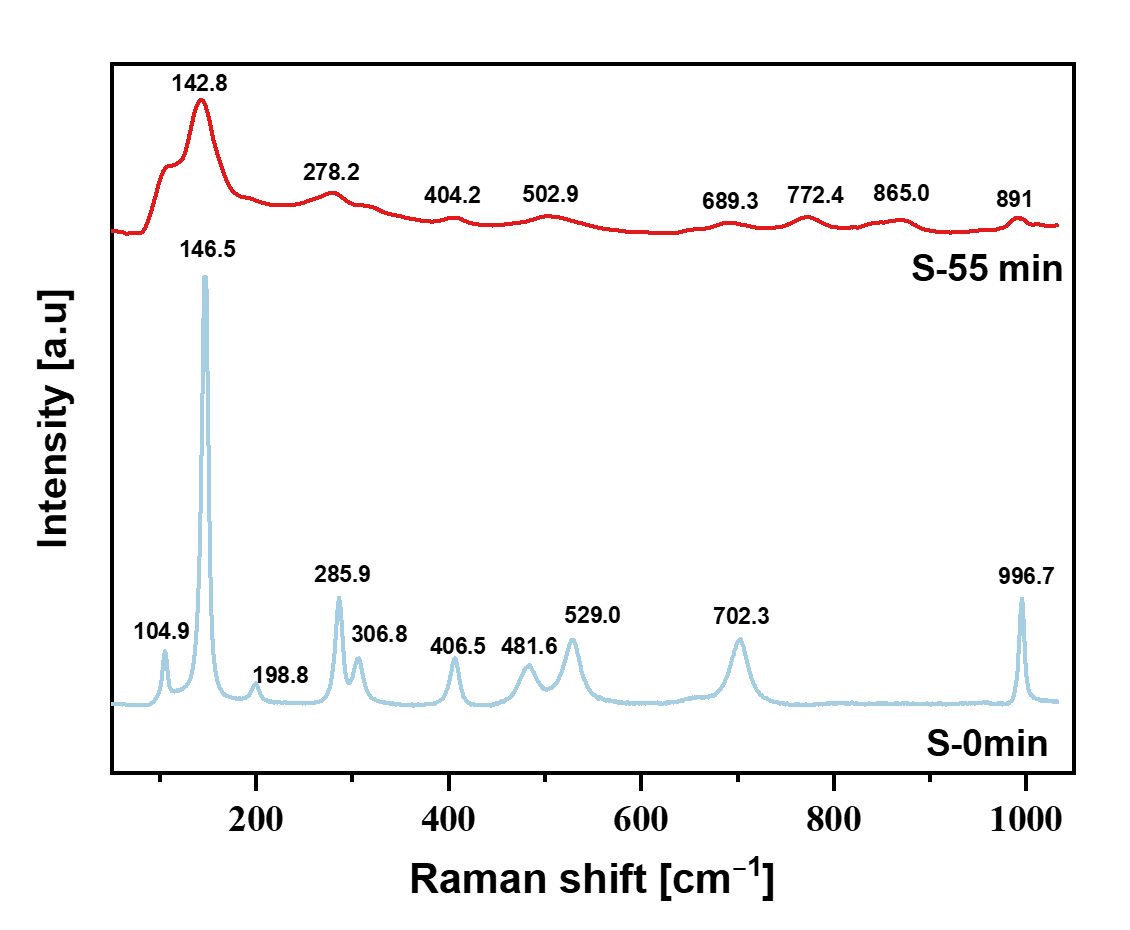


**Figure S7.** Raman spectra of S-0 and S-55 min with labeled peak positions. The Raman spectrum of S-0 min shows a set of peaks at 104.9, 146.5, 198.8, 285.9, 306.8, 406.5, 482.6, 529.0, 702.3, and 996.7 cm^−1^, with the following symmetry representations: 7A_g_ + 3B_1g_ + 7B_2g_ + 4B_3g_.


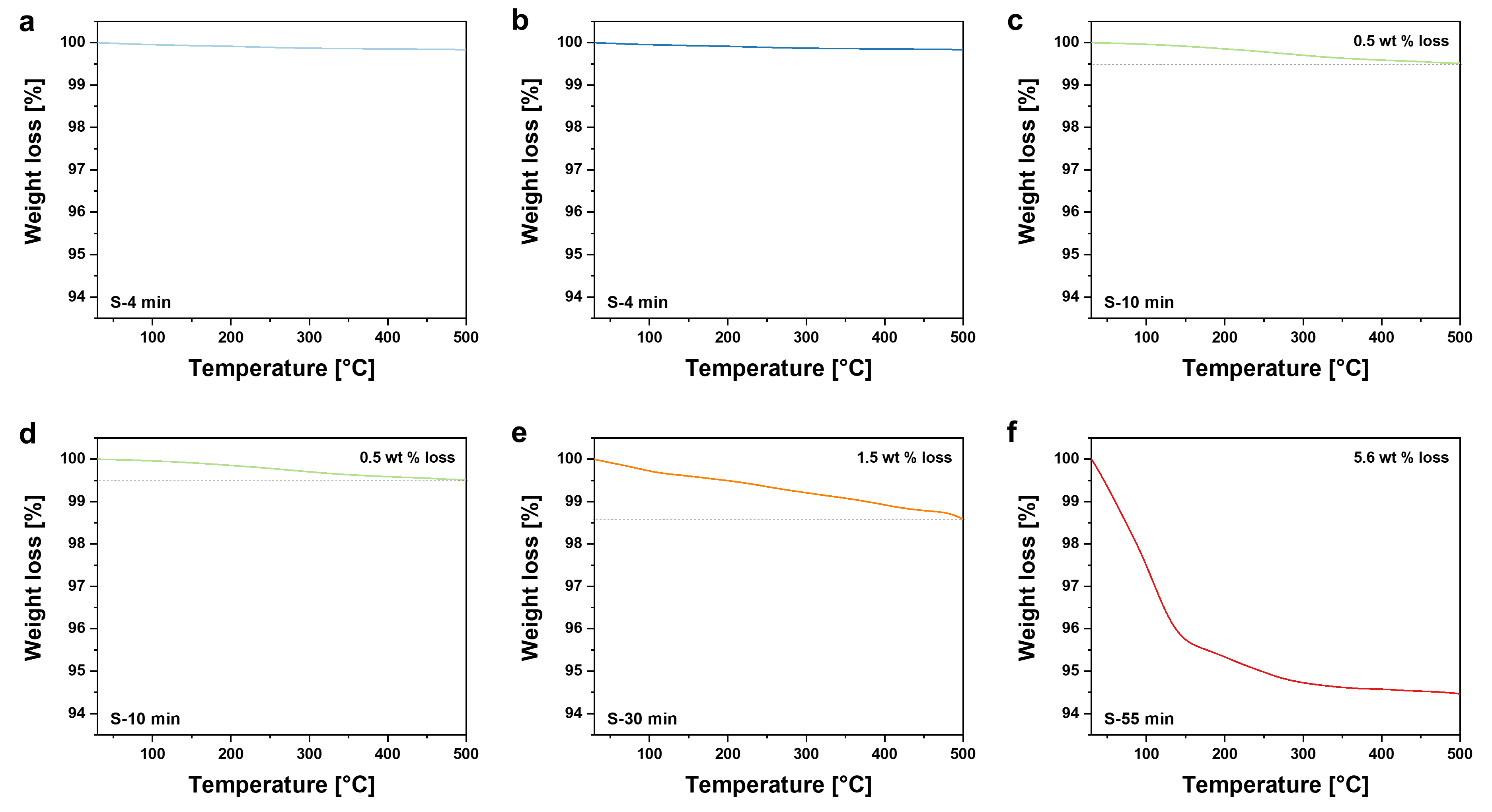


**Figure S8.** TGA of vanadate samples synthesized at various time points. All samples were vacuum-dried for 12 h prior to measurement to remove surface-adsorbed water.

a) S-0 min. b) S-4 min. c) S-10 min. d) S-15 min. e) S-30 min. f) S-55 min.


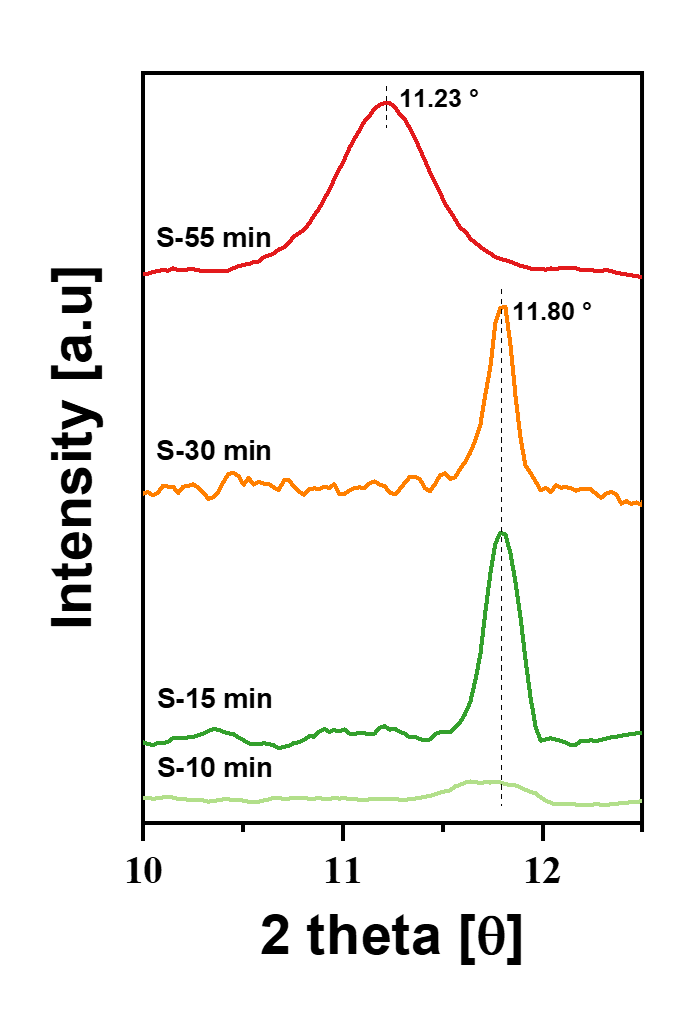


**Figure S9.** XRD patterns of S-10, S-15, S-30, and S-15, showing peak shifts at 10° and 13°.


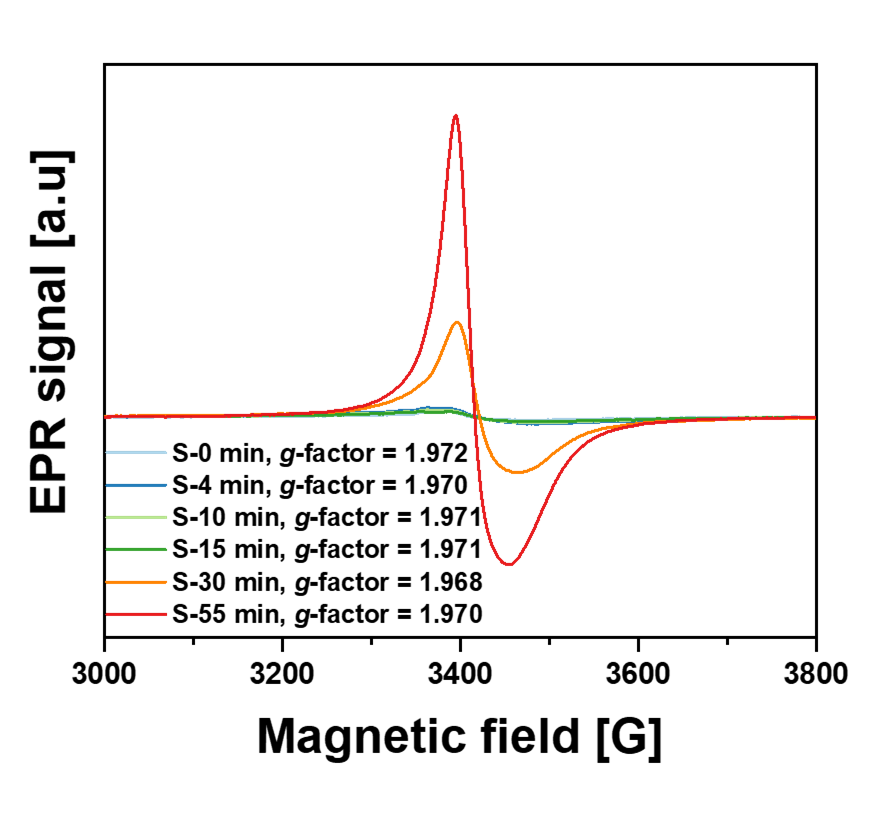


**Figure S10.** EPR spectra of vanadate samples synthesized at various time points. An isotropic signal with a g-factor of 1.97 was observed in all samples, indicating the presence of V^4+^ species. The EPR signal intensity progressively increased with synthesis time, reaching a maximum at S-55 min. This trend suggests that extended K^+^ intercalation weakens V–O bonds and promotes the partial reduction of V^5+^ to V^4+^.


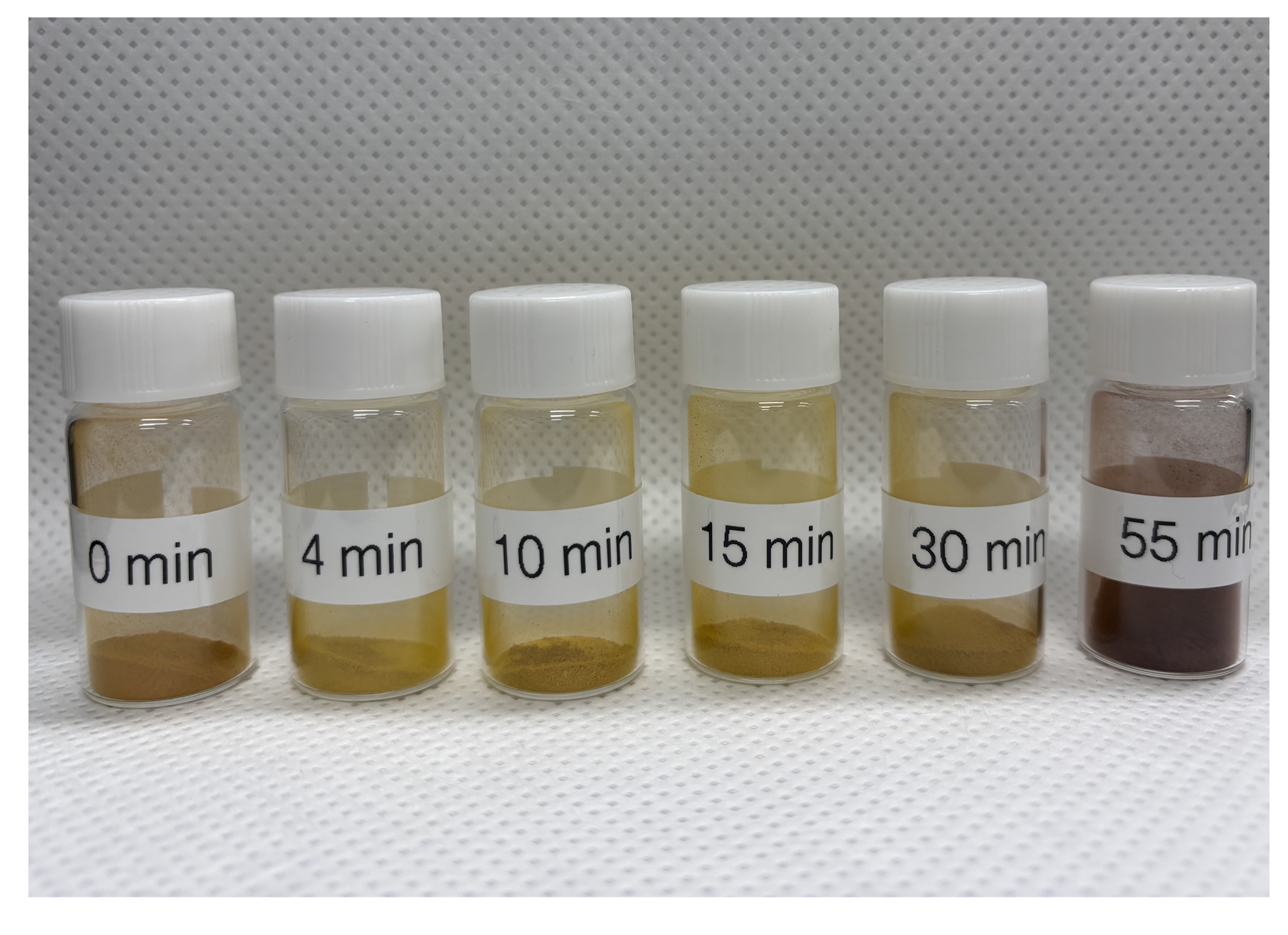


**Figure S11.** Progressive color change of vanadium oxide with synthesis time.


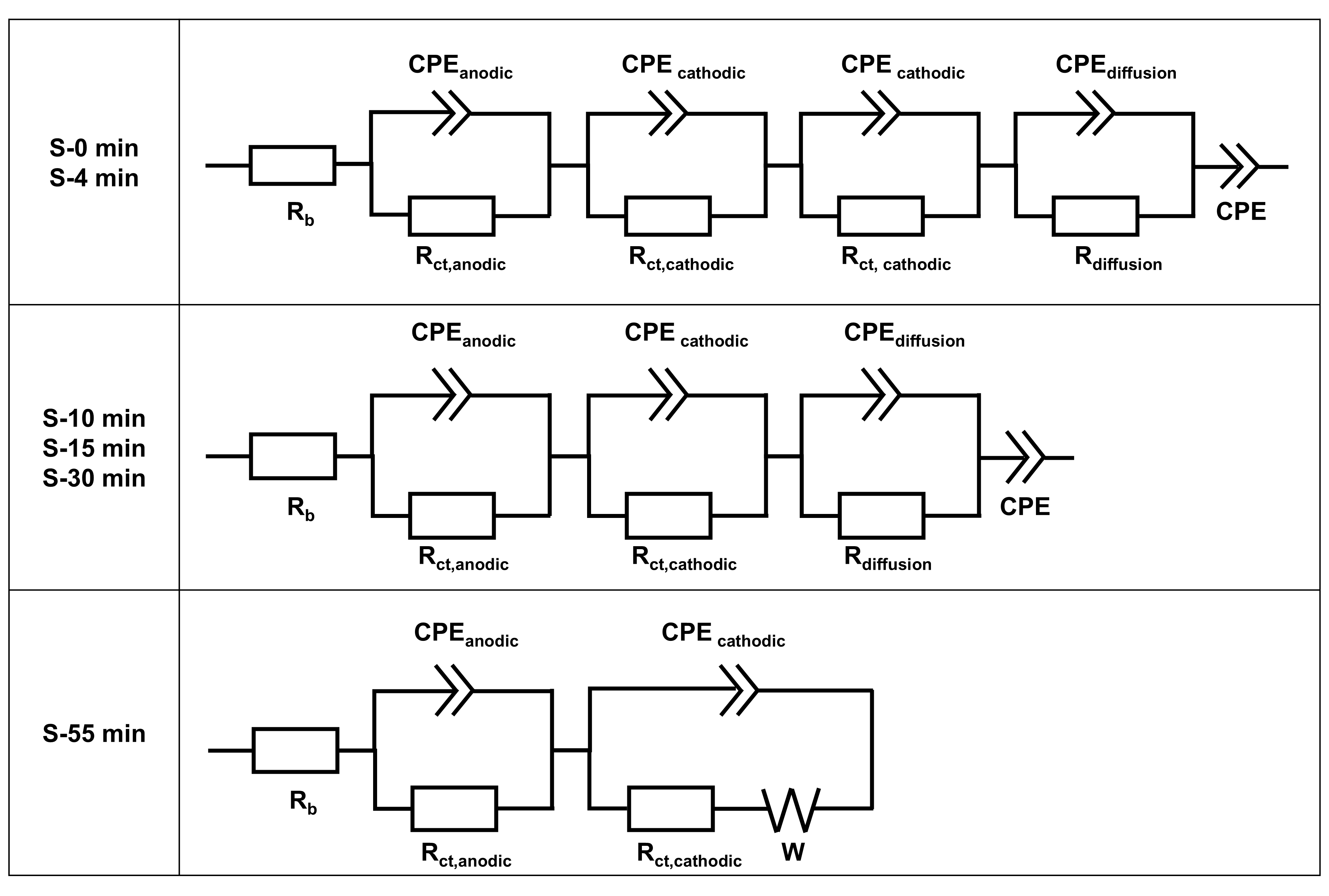


**Figure S12.** Equivalent circuit for nyquist plot fitting, with each R-C component representing charge transfer and diffusion processes. Each circuit element corresponds to a specific electrochemical process, including electrolyte resistance (R_b_), charge transfer at the anodic and cathodic, and diffusion characteristics. As the synthesis progresses, the number of R–CPE elements in the equivalent circuit decreases. This reduction in R–CPE elements indicates that the reaction kinetics have improved, leading to more homogeneous and faster interfacial processes. Starting from S-10 min, the enhanced charge-transfer kinetics lead to a merging of previously distinct responses, resulting in a single, well-defined semicircle in the Nyquist plots. This evolution corresponds to the progressive formation of the KVO phase, which improves Zn^2+^ transport and reduces interfacial resistance.


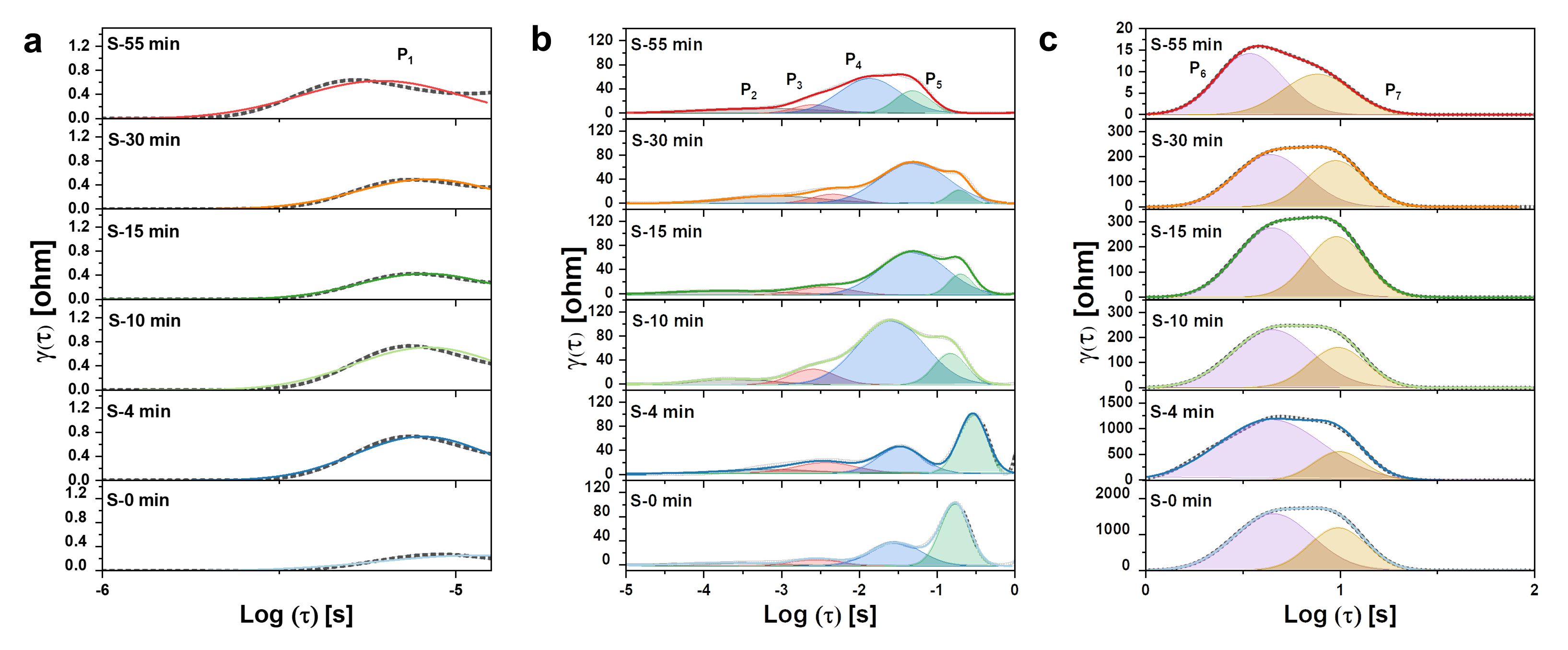


**Figure S13.** Deconvoluted DRT profiles. a) Bulk region. b) Charge transfer region. c) Diffusion region.

P_1_: Electrolyte Resistance.

P_2_: Charge Transfer at the SEI.

P_3_: Anodic Charge Transfer.

P_4_: Cathodic Charge Transfer.

P_5_: Cathodic Charge Transfer.

P_6_: Diffusion at the Electrode/Electrolyte Interphase.

P_7_: Bulk Diffusion Within the Cathode.


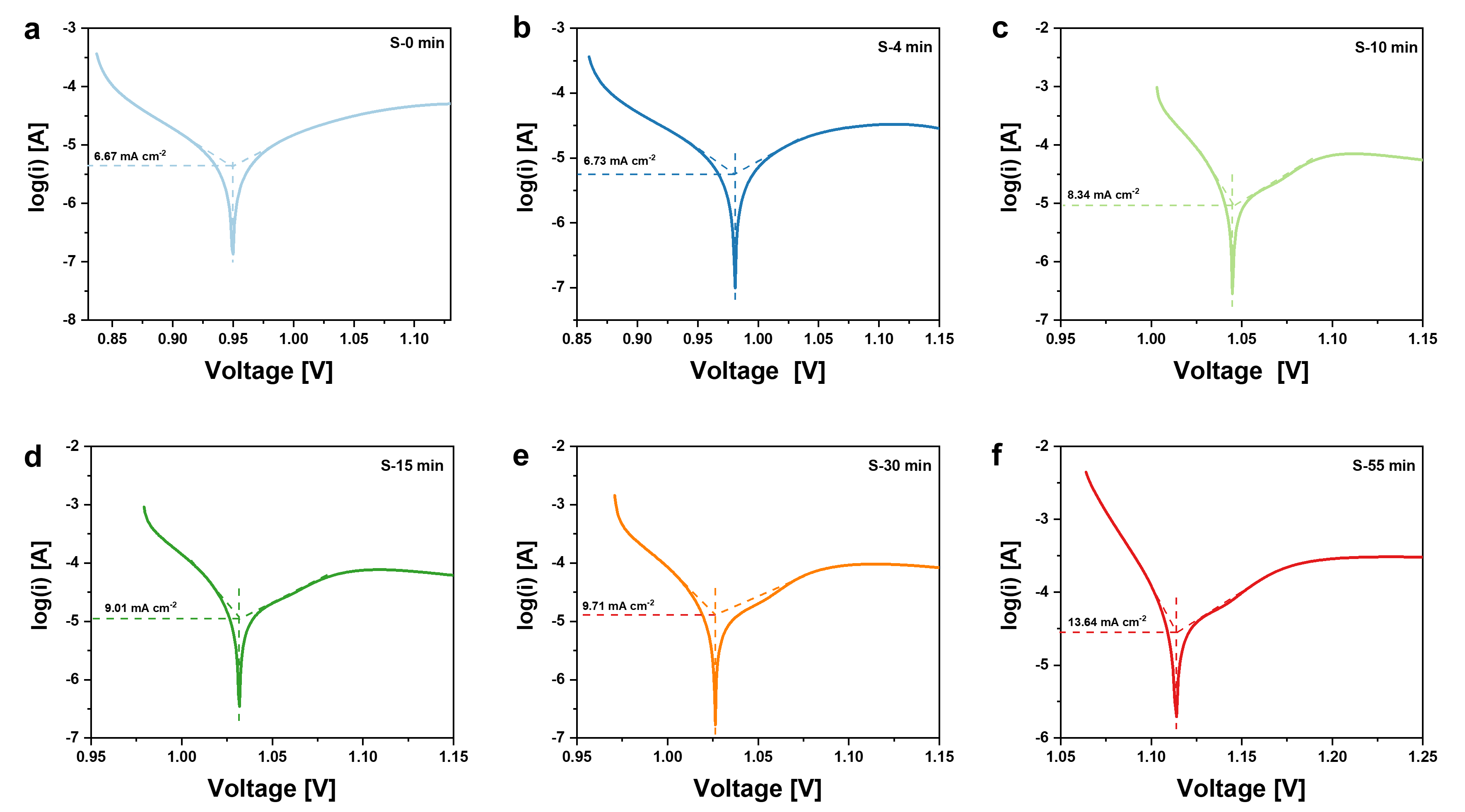


**Figure S14.** Tafel plot and *i_0_*. The *i₀* values were extracted by extrapolating the linear Tafel region to the equilibrium potential, where the reflects the activation overpotential required for the electrochemical reaction.

a) S-0 min. b) S-4 min. c) S-10 min. d) S-15 min. e) S-30 min. f) S-55 min.

**
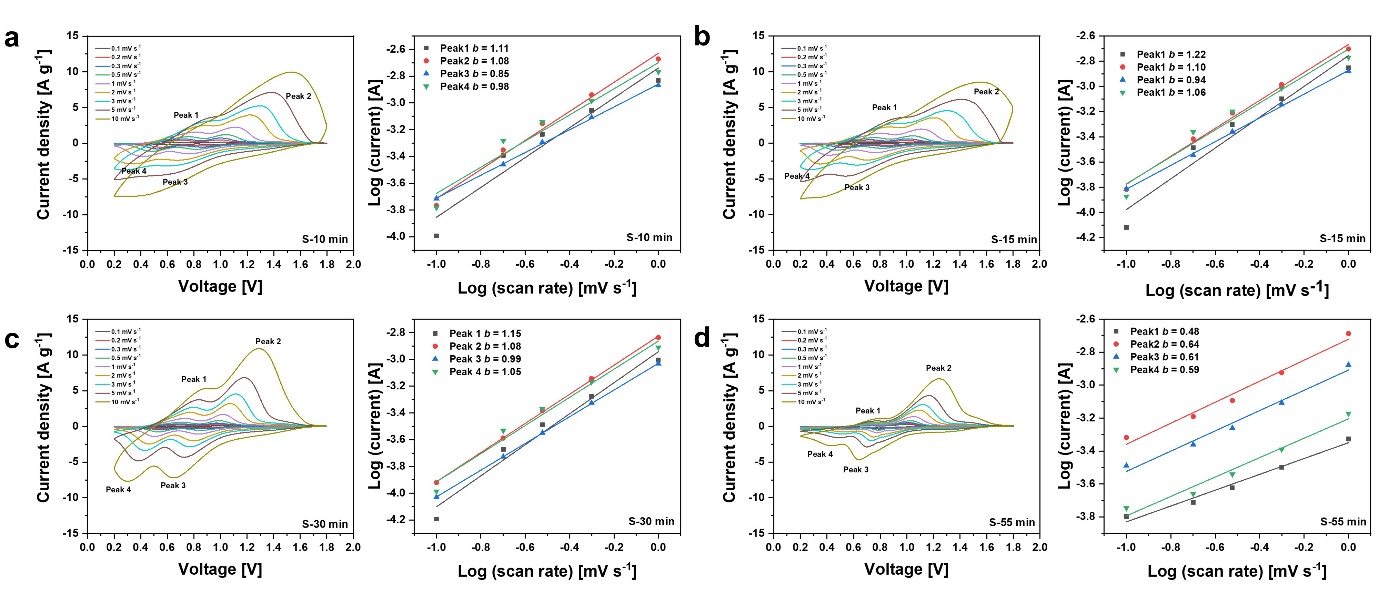
**

**Figure S15.** Cyclic voltammogram with various scan rates and corresponding *b*-value. a) S-10 min. b) S-15 min. c) S-30 min. d) S-55 min. At certain peaks, the *b*-value exceeded 1 or fell below 0.5. This deviation arises from the ohmic drop between the cathode and the Zn metal anode, which distorts the CV current–potential response. *b*-value analysis was not conducted for the early stage due to sluggish reaction kinetics, which resulted in indistinct CV signals and hindered precise quantification.

**
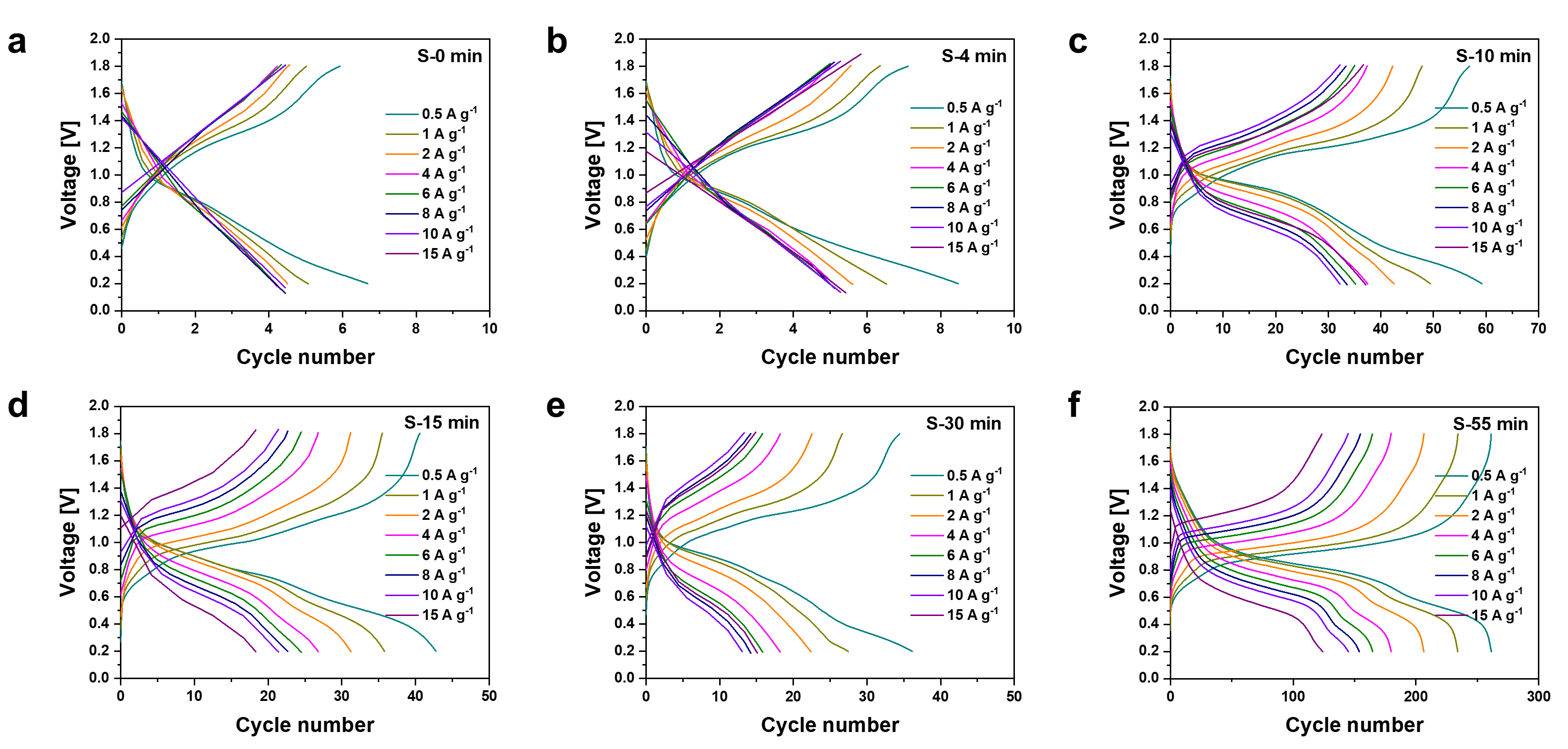
**

**Figure S16.** GCD profile with different current densities. a) S-0 min. b) S-4 min. c) S-10 min. d) S-15 min. e) S-30 min. f) S-55 min.


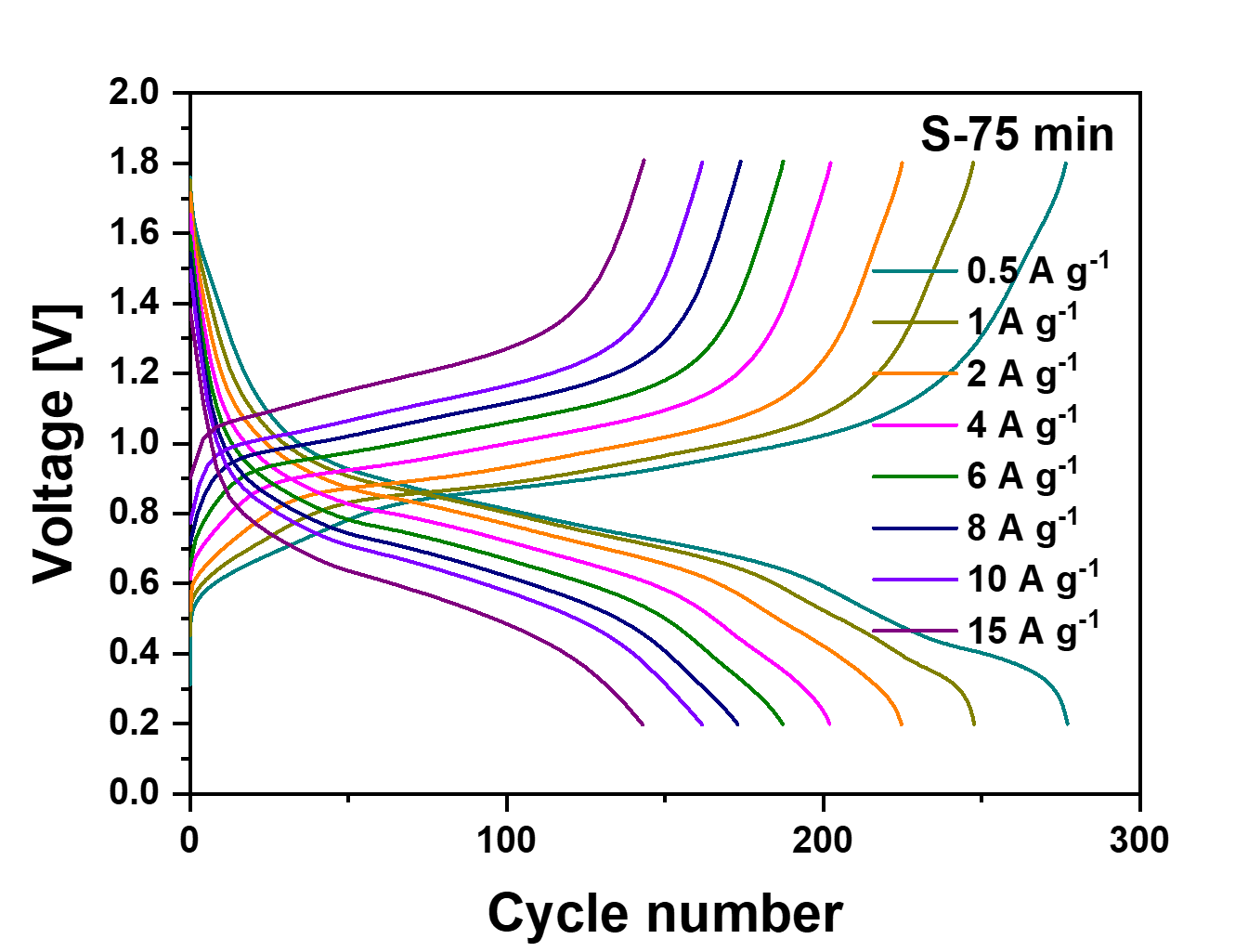


**Figure S17.** GCD profile with different current densities of S-75 min.


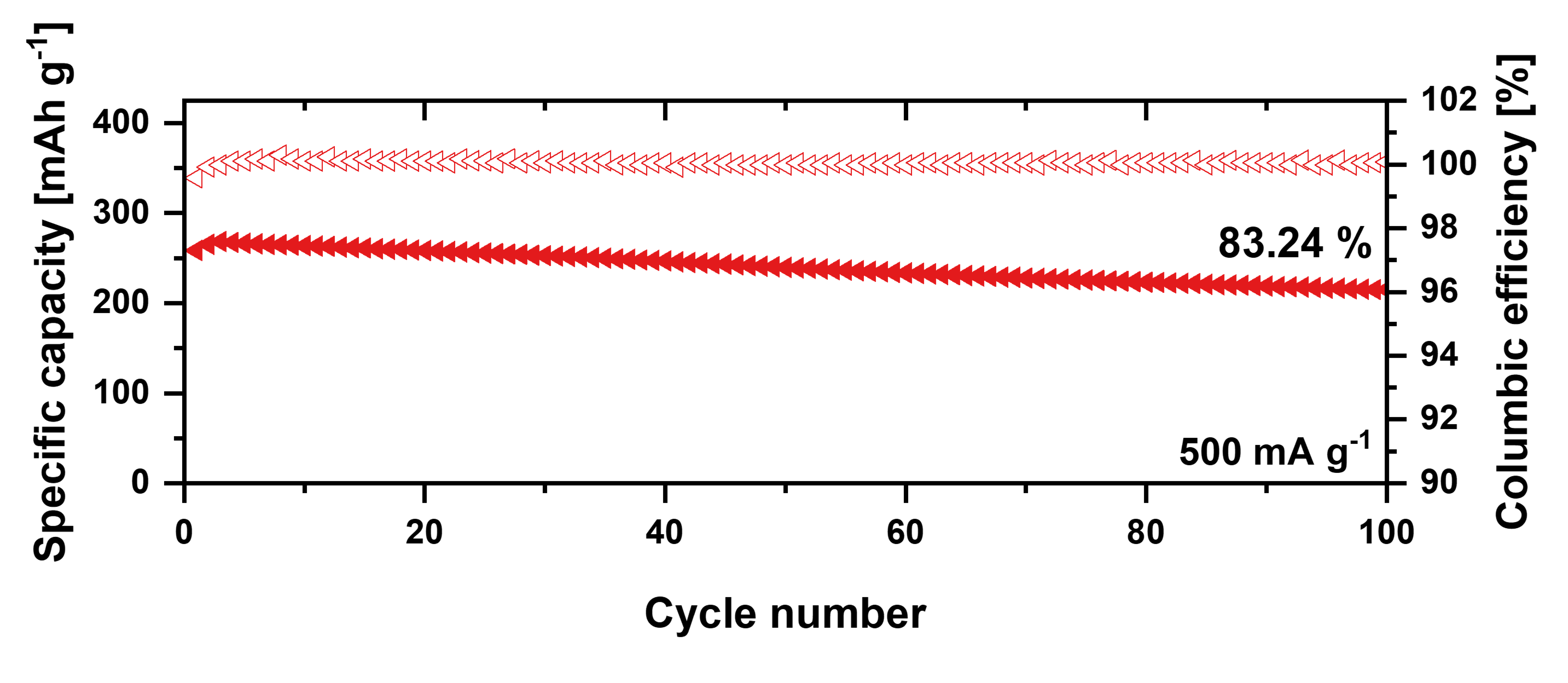


**Figure S18.** Cycle performance of S-55 min. at 500 mA g^-1^.


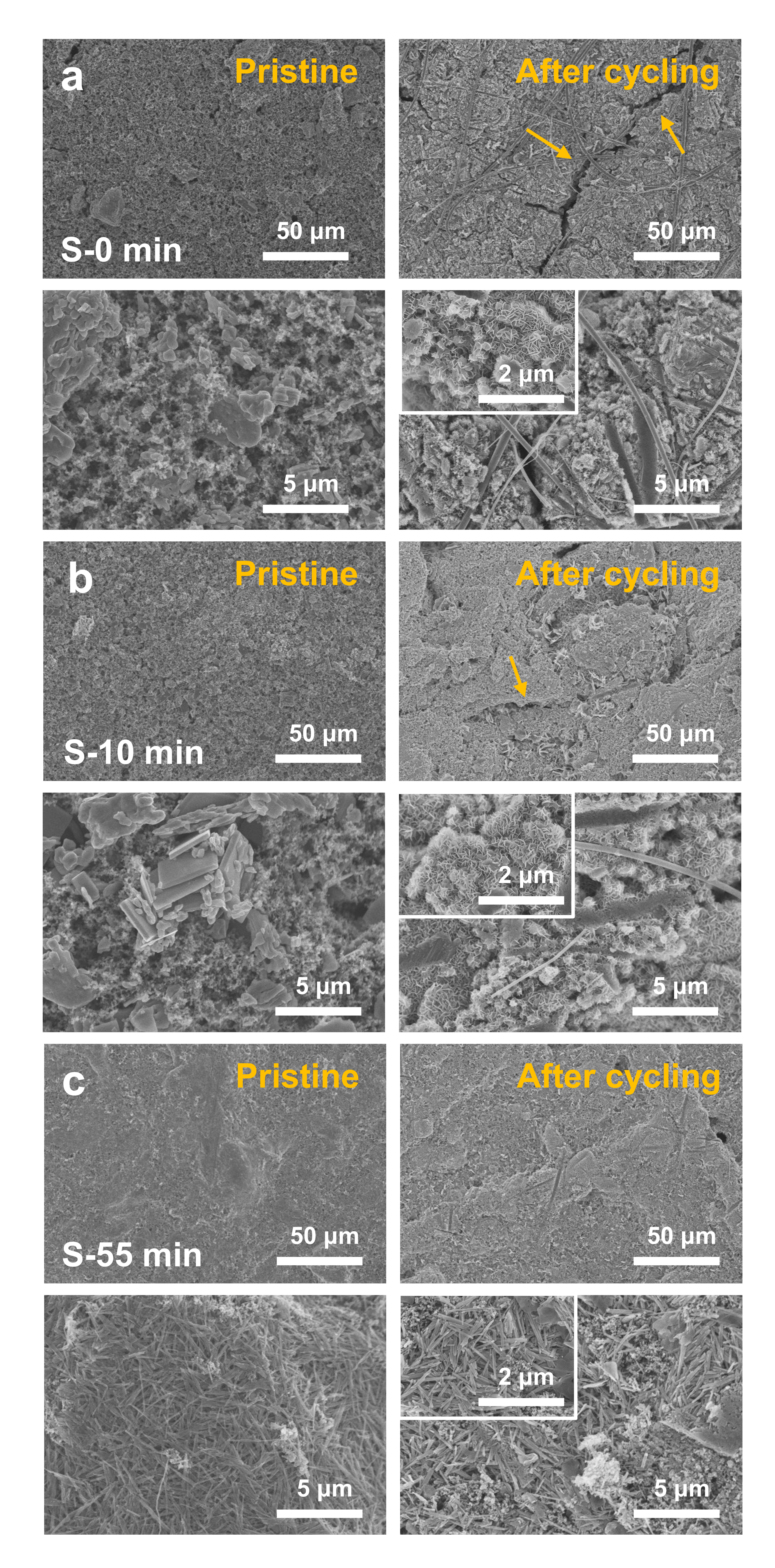


**Figure S19.** SEM images of S-0, S-10, and S-55 min electrodes before and after 100 cycles. a) S-0 min. b) S-10 min. c) S-55min. The post-cycling images reveal significant particle agglomeration and the formation of fine nanoflower-like structures on the surfaces of the S-0 and S-10 min. These nanoflowers are attributed to irreversibly formed zinc salt byproducts, which are commonly accompanied by vanadium dissolution into the aqueous electrolyte during cycling.


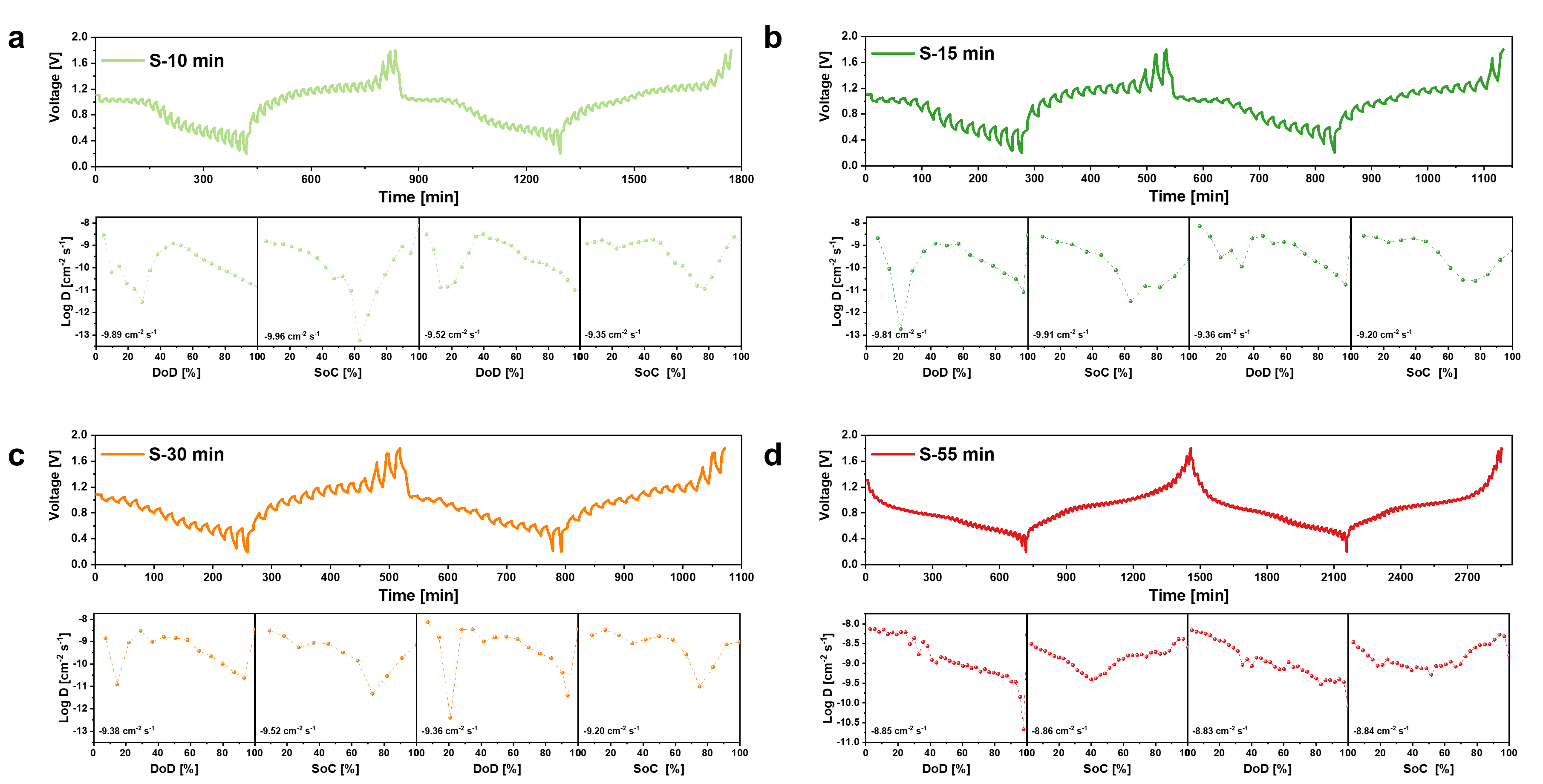


**Figure S20.** GITT prolife and *D_Zn_^2+^* during the initial two cycles.

a) S-10 min. b) S-15 min. c) S-30 min. d) S-55 min.


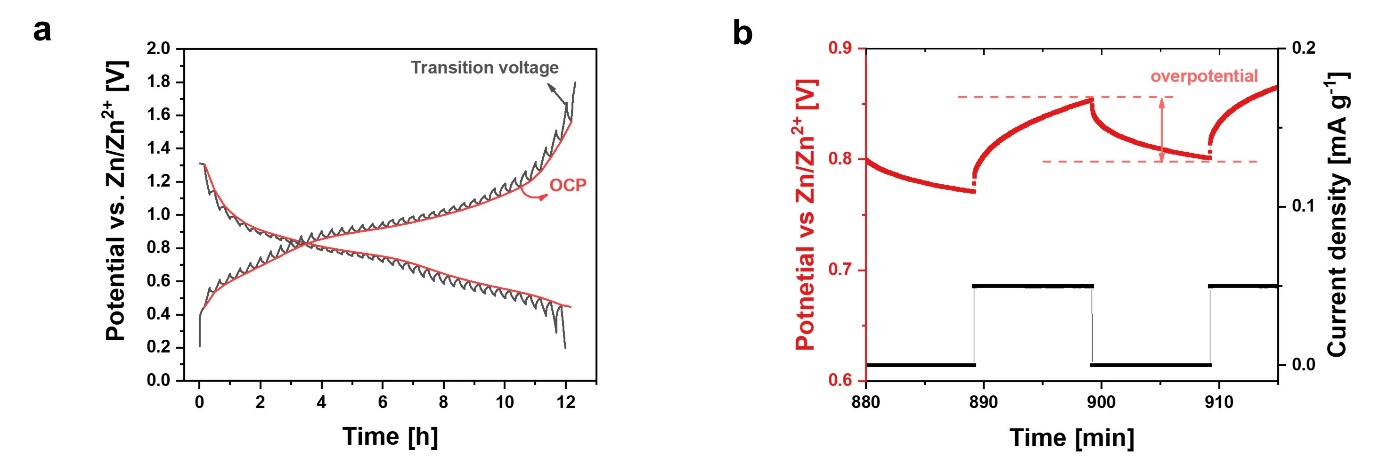


**Figure S21.** a-b) OCPs measured at the end of each relaxation period and *η* between OCP and transition voltage.

**
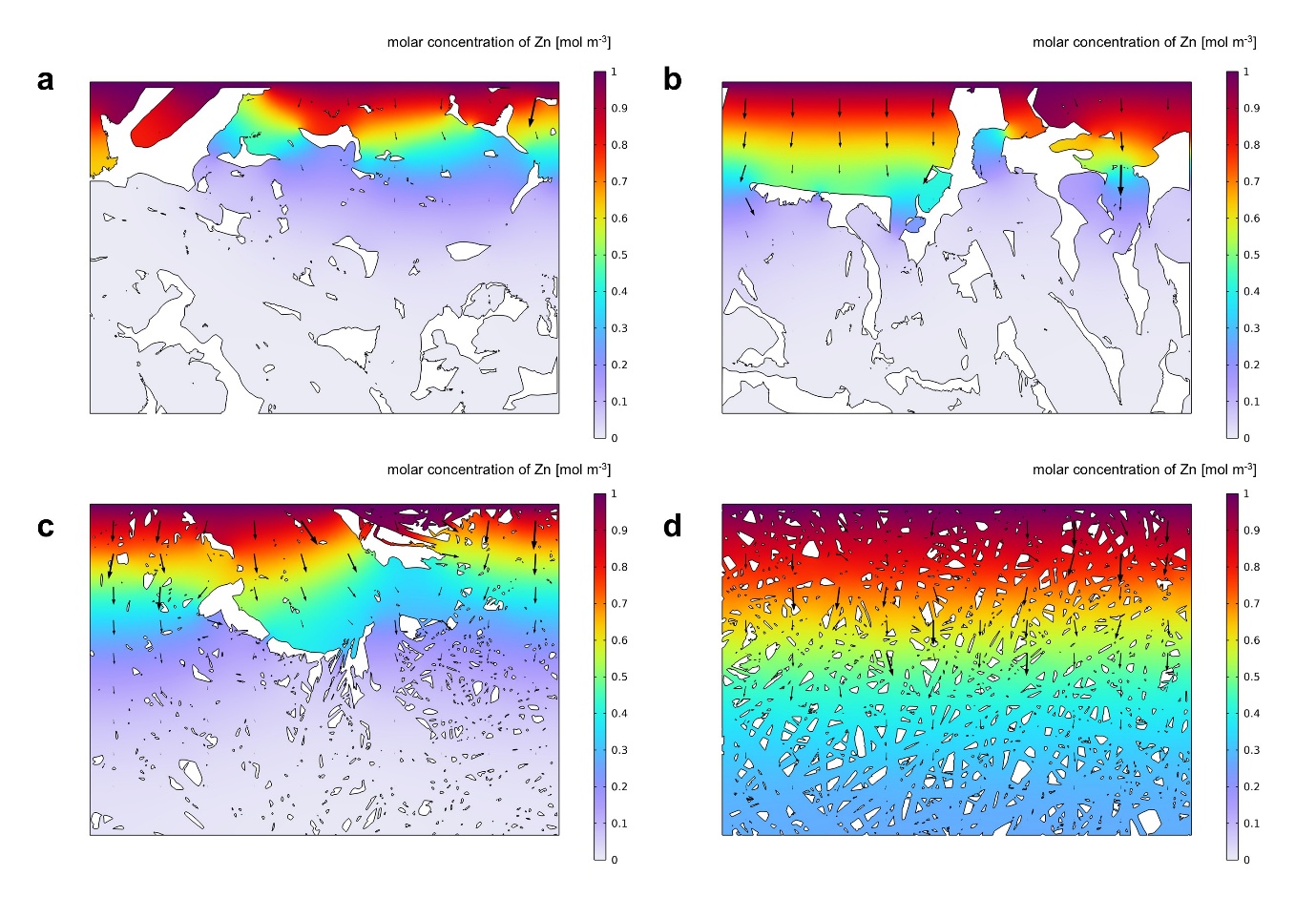
**

**Figure S22.** COMSOL simulation of Zn^2+^ diffusion within the electrode. The geometry was extracted from SEM image and *D_Zn_^2+^* was applied from GITT measurements. A fixed boundary concentration of 1 mol·m^-3^ was set to simulate Zn^2+^ diffusion into the electrode. a) S-10 min. b) S-15 min. c) S-30 min. d) S-55 min.

**
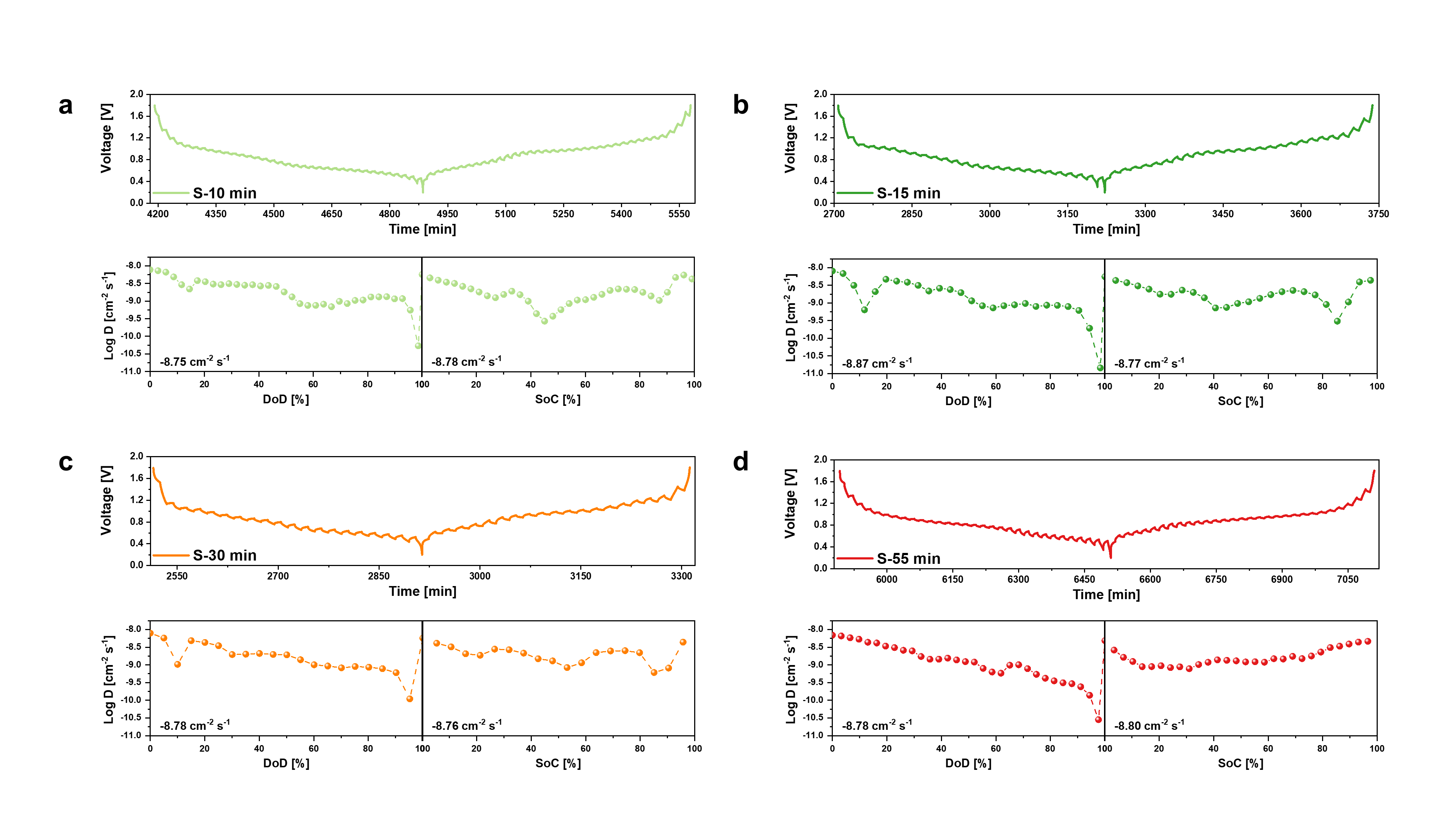
**

**Figure S23.** GITT prolife and *D_Zn_^2+^* at fifth cycles.

a) S-10 min. b) S-15 min. c) S-30 min. d) S-55 min.


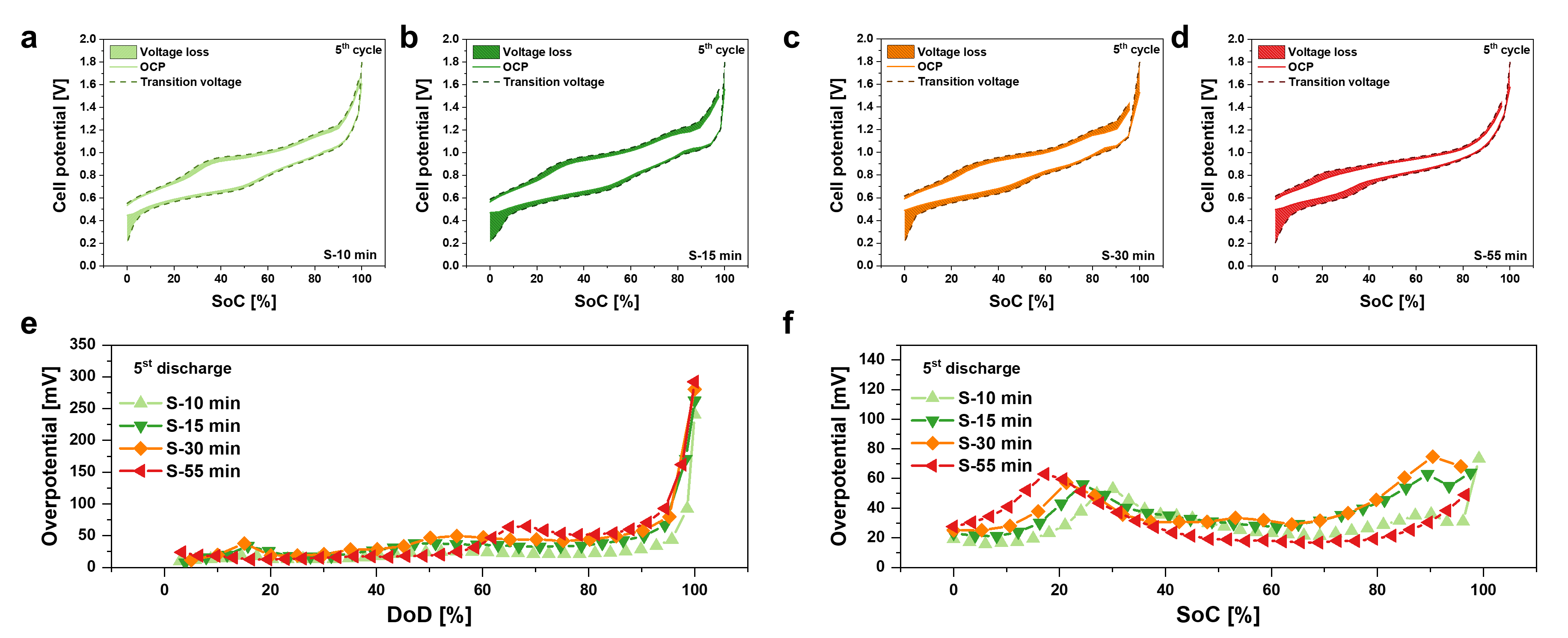


**Figure S24.** Variation of *η* with SoC. Voltage loss indicated by transient voltage and OCP during charge-discharge cycling. a) S-10 min. b) S-15 min. c) S-30 min. d) S-55 min. e-f) variation of overpotential during discharge and charge.


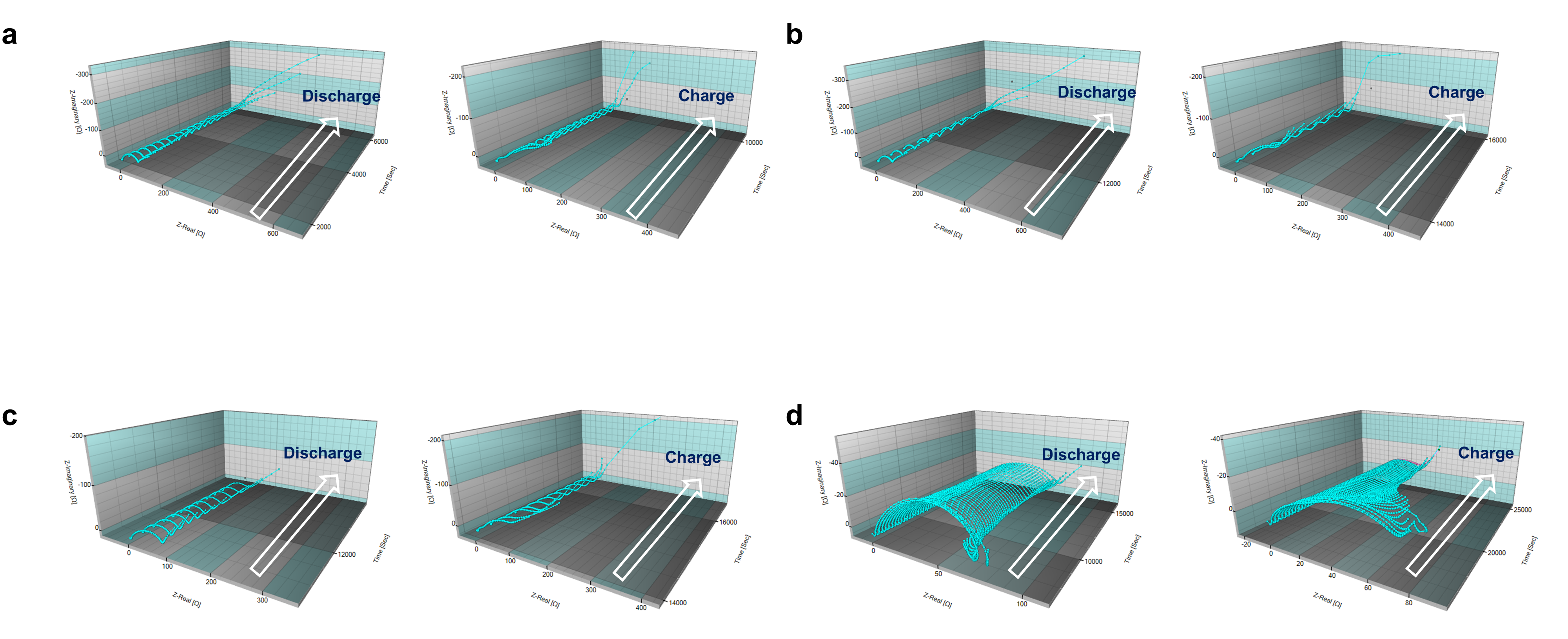


**Figure S25.** 3D Nyquist diagram composed of a real axis, an imaginary axis, and a time axis during 1^st^ cycle, with the left side corresponding to discharge and the right side to charge.

a) S-10 min. b) S-15 min. c) S-30 min. d) S-55 min.


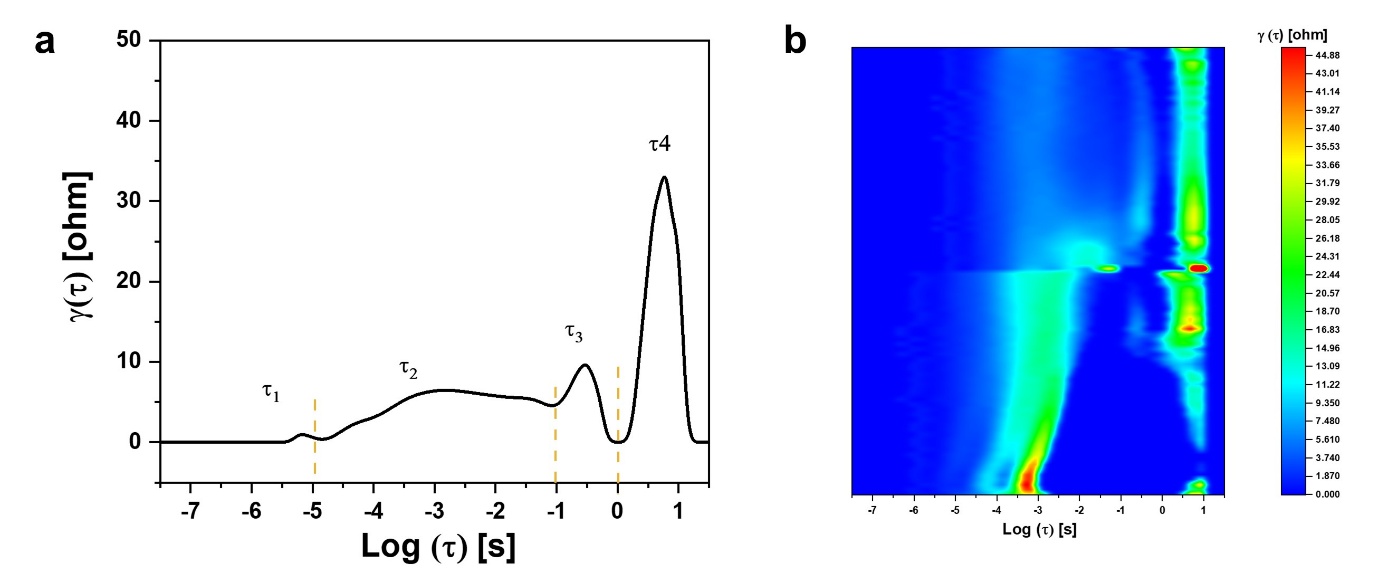


**Figure S26.** DRT analysis to decouple the processes governing the Nyquist plot. a) DRT profile highlighting four major regions. b) Contour plots of corresponding DRT

τ_1_: Bulk impednace (intrinsic impedance of the electrolyte)

τ_2_: Charge transfer (associated with charge transfer kinetics at anode/electrolyte interphase and cathode/electrolyte interphase)

τ_3_: Interfacial diffusion (Zn^2+^ accumulation at the electrode/electrolyte interphase)

τ_4_: Zn^2+^ diffusion within the cathode

**
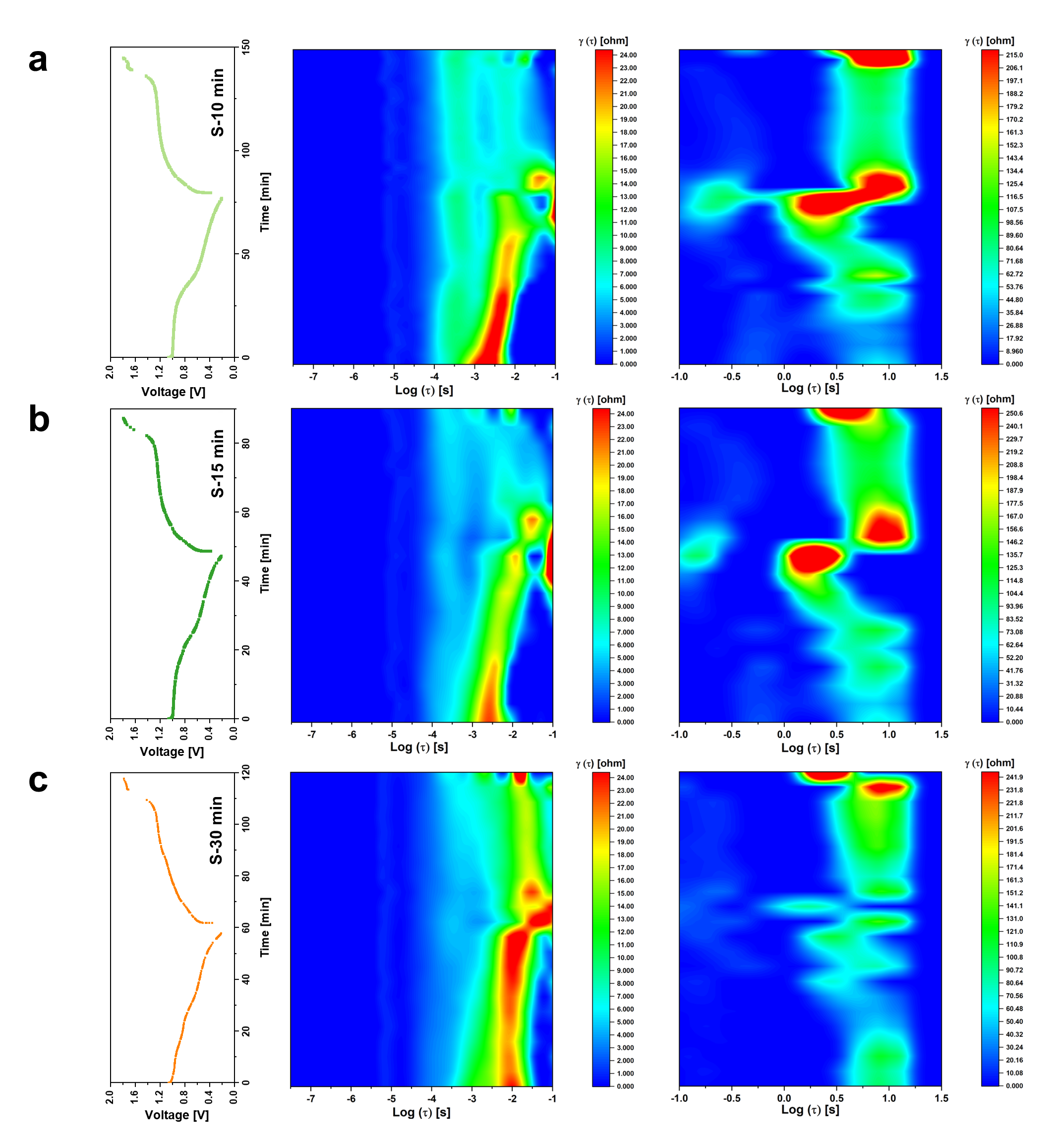
**

**Figure S27.** Contour plots of corresponding *operando*-DRT during 1^st^ cycle.

a) S-10 min. b) S-15 min. c) S-30 min.


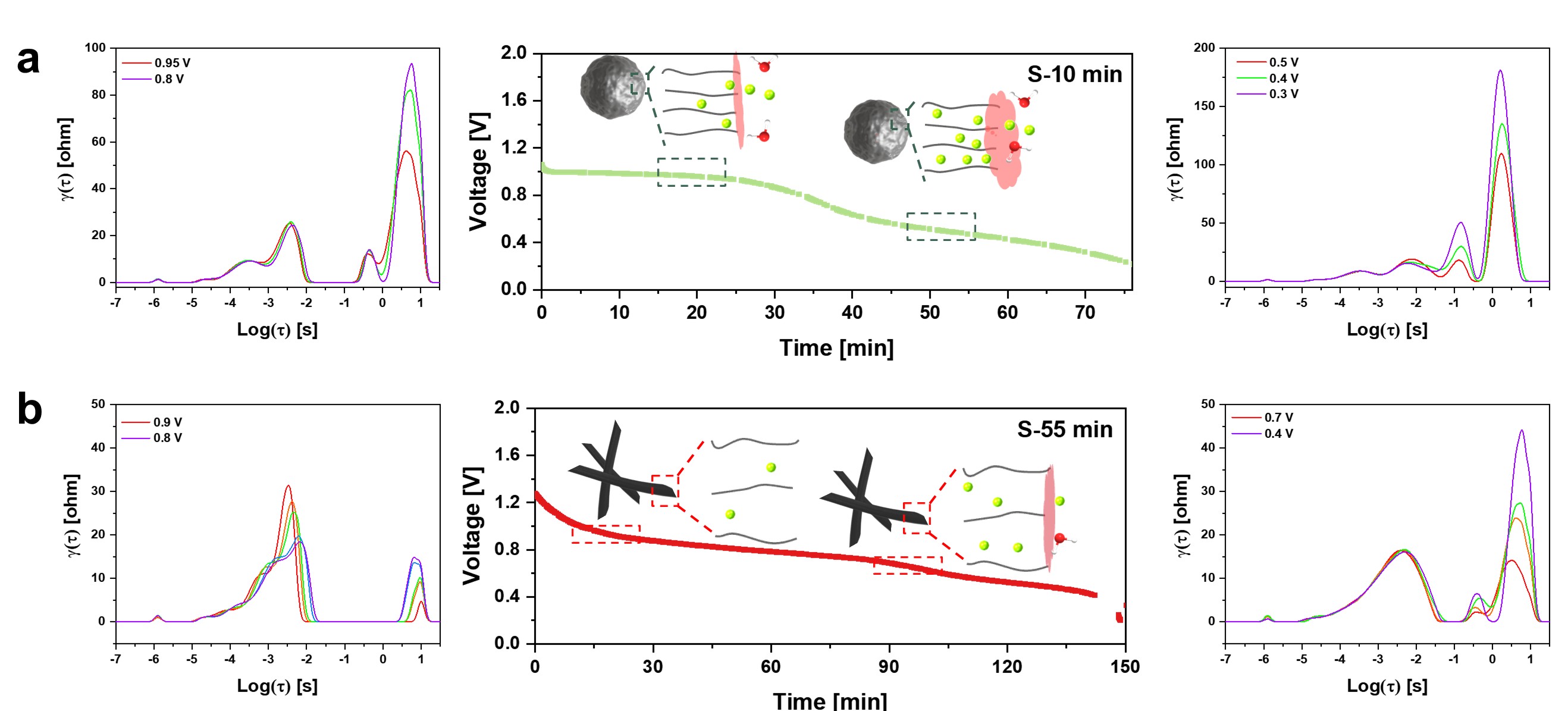


**Figure S28.** *Operando*-DRT profile at stage 1 and stage 2. a) S-10 min. b) S-55 min.


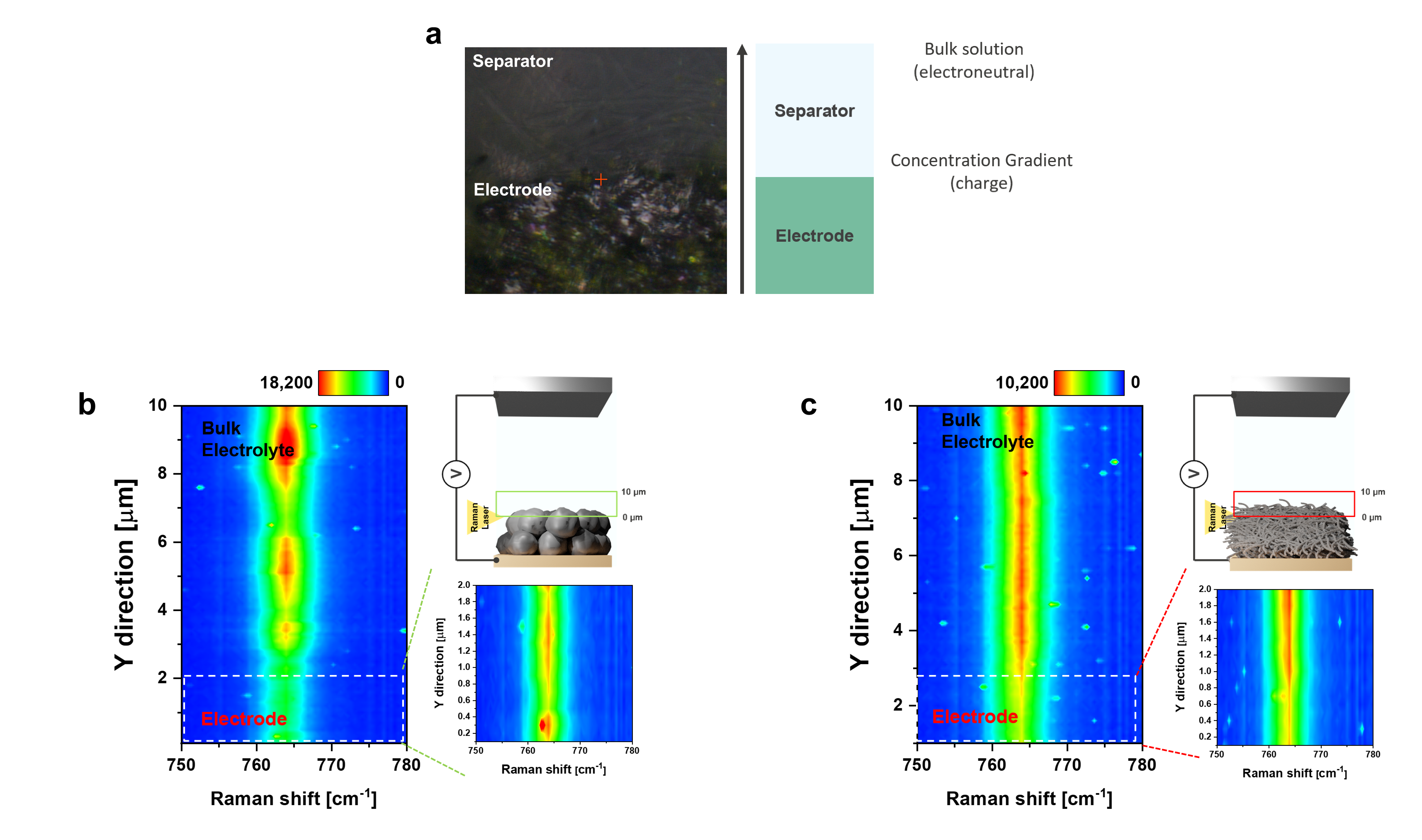
**Figure S29.** *Operando* cross-sectional Raman mapping analysis of interfacial ion distribution across the electrode/electrolyte interface. a) Schematic and optical image showing the Raman scanning direction along the Y-axis, from the electrode surface toward the separator. b) Raman mapping of the S-10 min. c) Raman mapping of the S-55 min.


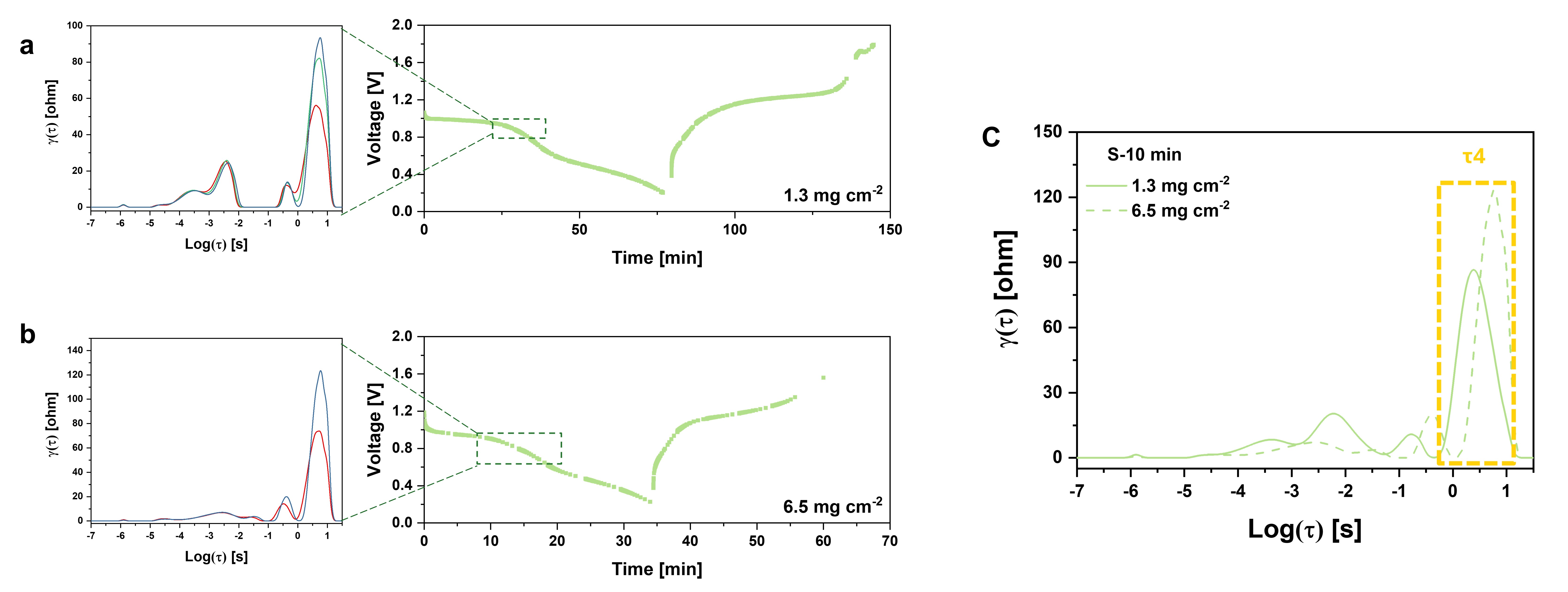


**Figure S30.** *Operando-*DRT analysis of S-10 min under different areal mass loadings during the first cycle. a) 1.3 mg cm^-2^. b) 6.5 mg cm^-2^.


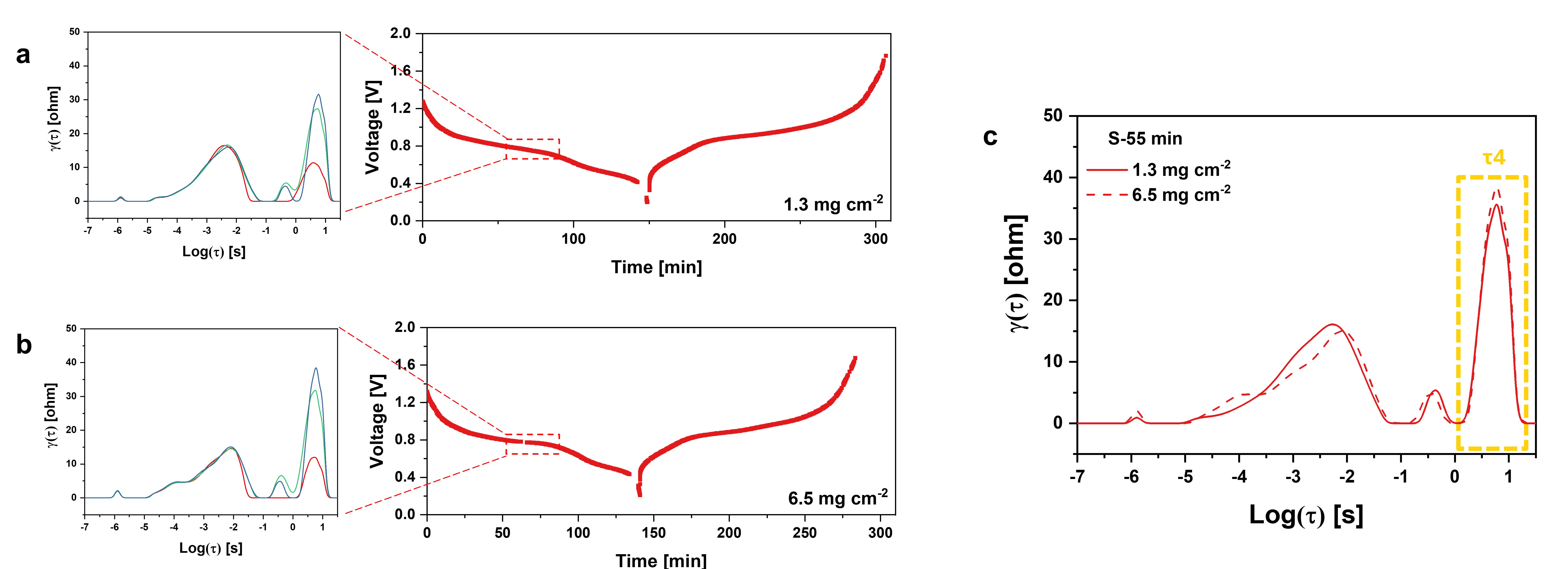


**Figure S31.** *Operando-*DRT analysis of S-55 min under different areal mass loadings during the first cycle. a) 1.3 mg cm^-2^. b) 6.5 mg cm^-2^.


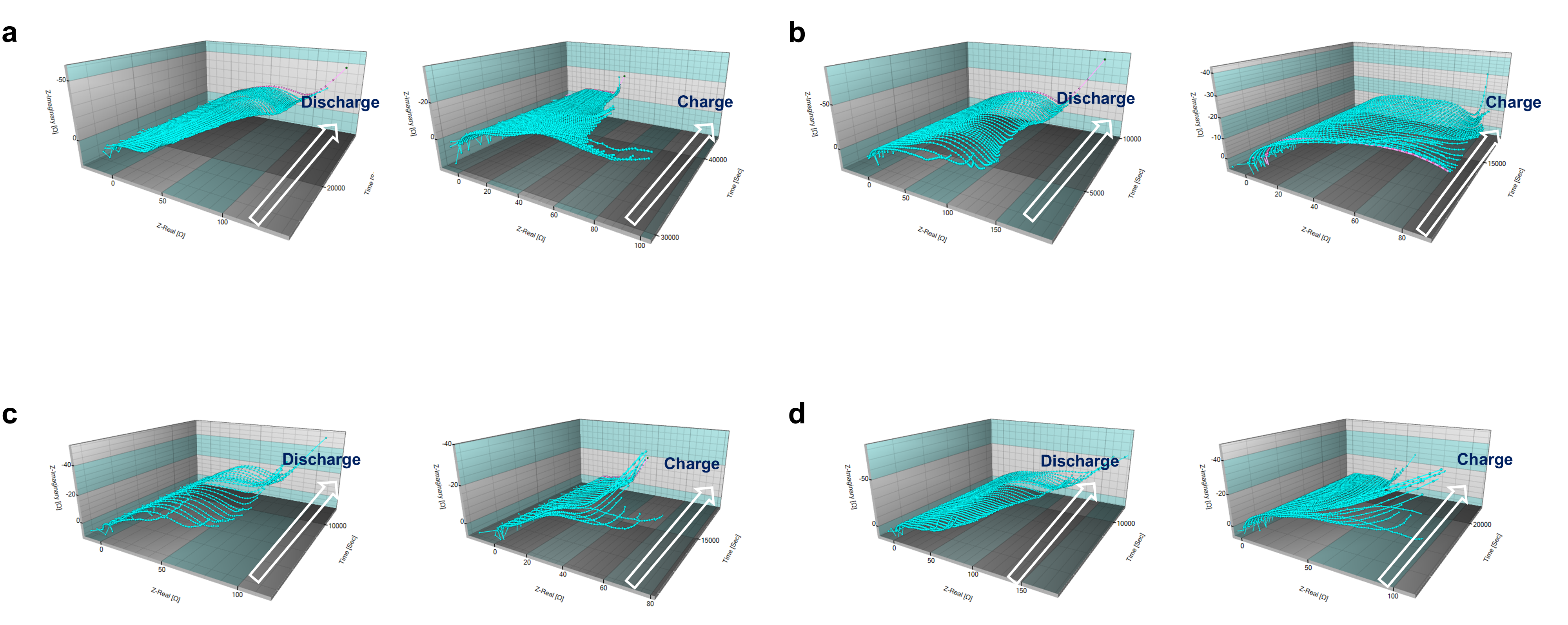


**Figure S32.** 3D Nyquist diagram composed of a real axis, an imaginary axis, and a time axis at 5^th^ cycle, with the left side corresponding to discharge and the right side to charge.

a) S-10 min. b) S-15 min. c) S-30 min. d) S-55 min.


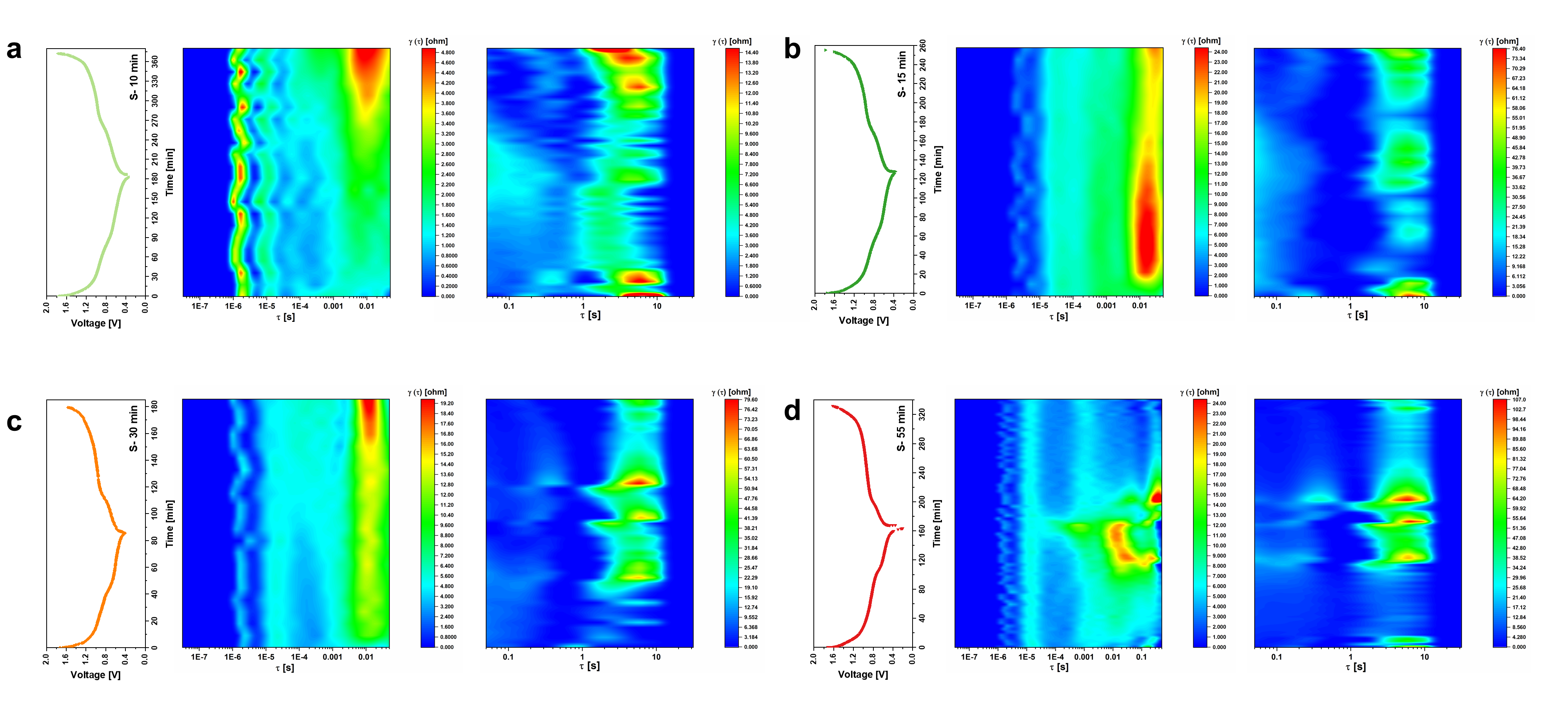


**Figure S33.** Contour plots of corresponding *operando*-DRT at 5^th^ cycle.

a) S-10 min. b) S-15 min. c) S-30 min.


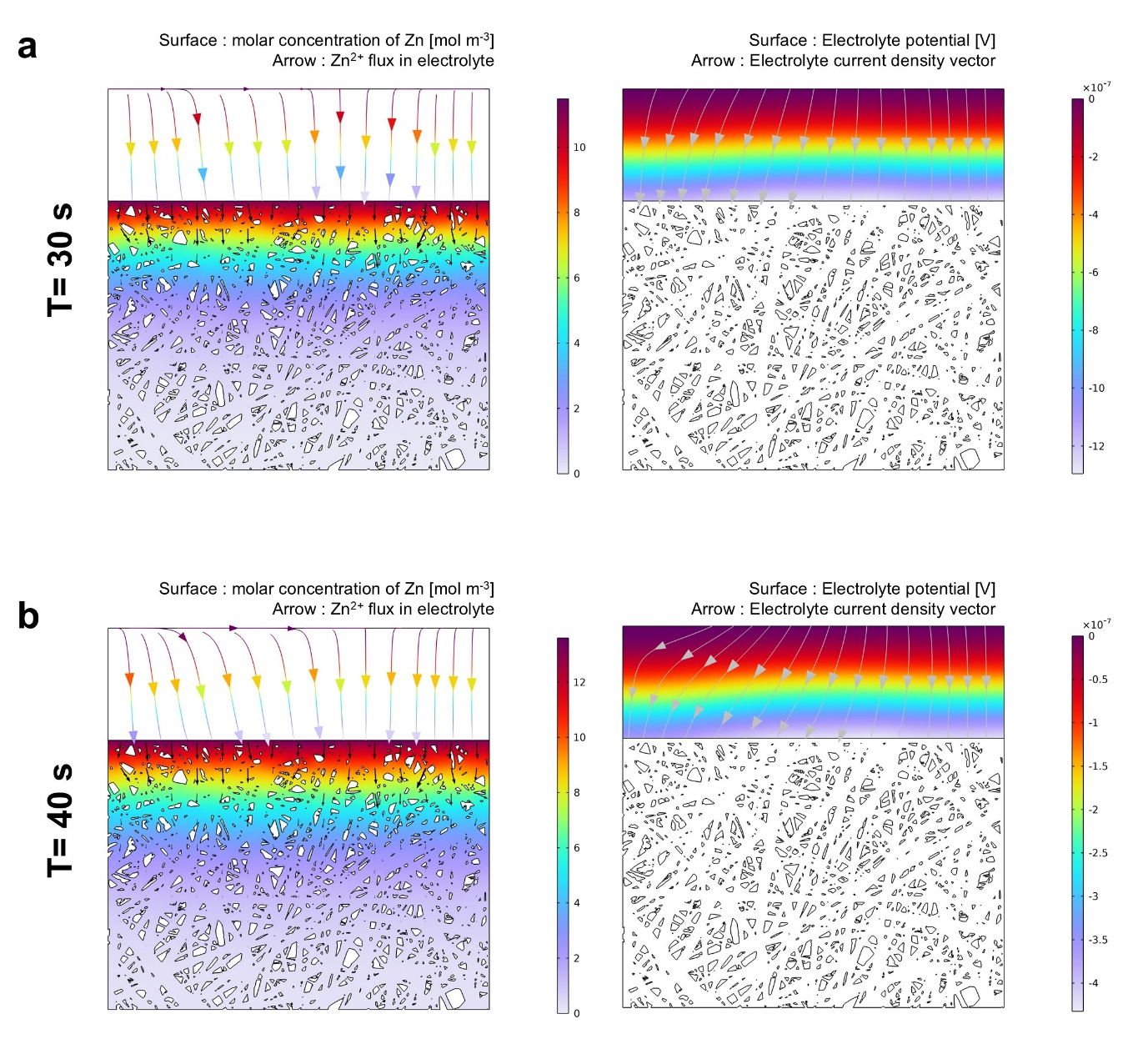


**Figure S34.** Time-dependent COMSOL simulation of Zn^2+^ flux (left) and electrolyte potential (right) at S-55. a) T=30 s. b) T= 40 s.
